# BCL-XL expression is essential for human erythropoiesis and engraftment of hematopoietic stem cells

**DOI:** 10.1038/s41419-019-2203-z

**Published:** 2020-01-06

**Authors:** Sehar Afreen, Sheila Bohler, Alexandra Müller, Eva-Maria Demmerath, Julia Miriam Weiss, Jonas Samuel Jutzi, Kristina Schachtrup, Mirjam Kunze, Miriam Erlacher

**Affiliations:** 1 Faculty of Medicine, Department of Pediatrics and Adolescent Medicine, Division of Pediatric Hematology and Oncology, University Medical Center Freiburg, University of Freiburg, Freiburg, Germany; 2 University of Freiburg, Freiburg, Germany; 3 Faculty of Medicine, Section of Molecular Hematology, Department of Medicine I, University Medical Center Freiburg, University of Freiburg, Freiburg, Germany; 4 Faculty of Medicine, Center for Chronic Immunodeficiency (CCI), Medical Center - University of Freiburg, Freiburg, Germany; 5 Faculty of Medicine, Department of Obstetrics and Gynecology, University Medical Center Freiburg, University of Freiburg, Freiburg, Germany; 6 German Cancer Consortium (DKTK), Freiburg, Germany and German Cancer Research Center (DKFZ), Heidelberg, Germany

**Keywords:** Haematological diseases, Translational research

## Abstract

The anti-apoptotic BCL-2 proteins (BCL-2, BCL-XL, MCL-1, A1, BCL-W) counteract apoptotic signals emerging during development and under stress conditions, and are thus essential for the survival of every cell. While the “BCL-2 addiction” of different cell types is well described in mouse models, there is only limited information available on the role of different anti-apoptotic BCL-2 proteins in a given human cell type. Here we characterize the role of BCL-XL for survival and function of human hematopoietic cells, with the aim to predict hematological side effects of novel BCL-XL-inhibiting BH3-mimetics and to identify hematological malignancies potentially responsive to such inhibitors. Earlier clinical studies have shown that the combined BCL-2/BCL-XL/BCL-W inhibitor, Navitoclax (ABT-263) induces severe thrombocytopenia caused by direct platelet demise and counteracted by increased megakaryopoiesis. In contrast, murine studies have reported important contribution of BCL-XL to survival of late erythroid cells and megakaryocytes. Using lentiviral knockdown, we show that the roles of BCL-XL for human hematopoietic cells are much more pronounced than expected from murine data and clinical trials. Efficient genetic or chemical BCL-XL inhibition resulted in significant loss of human erythroid cells beginning from very early stages of erythropoiesis, and in a reduction of megakaryocytes. Most importantly, BCL-XL deficient human hematopoietic stem cells and multipotent progenitors were reduced in numbers, and they showed a severely impaired capacity to engraft in mice during xenotransplantation. BCL-XL deficiency was fully compensated by BCL-2 overexpression, however, loss of its antagonist BIM did not result in any rescue of human erythroid or stem and progenitor cells. We thus conclude that novel and specific BCL-XL inhibitors might be efficient to treat malignancies of erythroid or megakaryocytic origin, such as polycythemia vera, acute erythroid leukemia, essential thrombocytosis or acute megakaryocytic leukemia. At the same time, it can be expected that they will have more severe hematological side effects than Navitoclax.

## Introduction

The BCL-2 family represents an integration hub for cell death and survival signals, where in simple terms the “cell fate” is determined by the balance between pro- and anti-apoptotic family members. Cell survival under steady-state conditions is ensured by the anti-apoptotic BCL-2 proteins, i.e. BCL-2, BCL-XL, MCL-1, A1, and BCL-W. They bind to the downstream pro-apoptotic effector proteins, BAX and BAK, thereby preventing their activation and multimerization. Lethal cell stress leads to transcriptional or posttranslational activation of the upstream pro-apoptotic BCL-2 proteins belonging to the BH3-only subgroup. Eight classical BH3-only proteins have been described, which include BIM, BMF, PUMA and BAD. They bind with varying affinities to one or more anti-apoptotic BCL-2 proteins, thereby displacing BAX and BAK. Consequently, BAX/BAK are activated and form large pores that lead to mitochondrial outer membrane permeabilization (MOMP), activation of the caspase cascade and apoptotic cell death^1,2^. While it became apparent that survival of every cell depends on the presence of at least one anti-apoptotic BCL-2 protein, the individual “BCL-2 addiction” of a given cell type has only been insufficiently described. Genetic mouse models revealed that mature lymphocytes and melanocytes depend on BCL-2 expression for survival, while MCL-1 is essential for successful nidation and survival of stem cells and early progenitors in hematopoietic system^3–5^. Not long ago, BCL-2 protein inhibitors, the so called BH3-mimetics, were found to be effective anticancer drugs. As a result, attention was drawn to the “BCL-2 addiction” of tumor cells across different entities^6^. Navitoclax (ABT-263), a BCL-2/BCL-XL/BCL-W inhibitor, showed strong effects against chronic lymphocytic leukemia (CLL), but also resulted in severe thrombocytopenia^7^. However, Navitoclax was successfully replaced by a specific BCL-2 inhibitor, Venetoclax (ABT-199), which showed fewer side effects and was approved in 2016 by FDA for treatment of CLL^8^. The deleterious effects of BCL-2 inhibition in human CLL and murine mature lymphocytes indicate that “BCL-2 addiction” is transmitted from the cell of origin to its malignant descendants. Nevertheless, a species-specific comparison of naïve human hematopoietic cells and human malignancies might provide even better correlations. Therefore, deeper insights into the “BCL-2 addiction” of individual human hematopoietic cells would be valuable to predict the efficacy of specific BH3-mimetics in hematological malignancies and foresee potential hematological side effects of such substances.

Here, we analyzed the role of BCL-XL (BCL2L1) in the human hematopoietic system. BCL-XL was the first identified BCL-2 homologue and represents one of two splicing variants of the *BCL-X* gene^9,10^. It binds to BIM, BMF, BAD, BIK, HRK, PUMA, tBID, and to BAX and BAK as well^11^. By shuttling BAX from mitochondria to cytosol, BCL-XL reduces BAX levels at mitochondria and apoptotic susceptibility of cells^12^. When overexpressed, BCL-XL (like BCL-2) prevents apoptosis caused by a plethora of stress signals. Endogenous BCL-XL is essential for normal embryogenesis and BCL-X deficient embryos die around E13 with increased apoptosis rates in post-mitotic immature neurons of brain, spinal cord and dorsal root ganglia^13^. Fetal livers showed massive apoptosis of hematopoietic progenitors, but generation of chimeric mice revealed that *Bcl-x*^−/−^ deficient hematopoietic stem and progenitor cells (HSPC) contributed to hematopoiesis and lymphopoiesis^13,14^. Similarly, *Bcl-x* deletion in adult murine hematopoietic cells impaired erythropoiesis but did not affect the HSPC compartment and myeloid differentiation^15^. Recent work suggests that in contrast to young hematopoietic stem cells (HSCs), senescent HSCs become increasingly dependent on BCL-2 and/or BCL-XL expression, as they are effectively cleared in aged mice by Navitoclax^16^. Different conditional, lineage-specific mouse models of *Bcl-x* deficiency further revealed its pivotal role in the survival of differentiated hematopoietic cells including mature megakaryocytes, terminal differentiation stages of erythropoiesis and macrophages^14,17–19^. Loss of *Bcl-x* deficient megakaryocytes and erythrocytes resulted in compensatory proliferation of their immature progenitors, indicating that BCL-XL addiction of murine hematopoietic cells increases with their differentiation^17,20^.

Navitoclax-induced thrombocytopenia revealed for the first time that programmed demise of platelets, albeit not being cells, depends on the intrinsic apoptosis machinery. BCL-XL abundance was shown to define platelet lifespan, and its inhibition by Navitoclax resulted in rapid platelet loss^21^. However, thrombocytopenia could be compensated by increased megakaryopoiesis. Other hematopoietic side effects of Navitoclax included anemia and neutropenia in some but not all patients^7,22^. These clinical observations suggested that BCL-XL plays a minor role in human than in murine hematopoiesis. However, observations made in patients treated with a combined BCL-2/BCL-XL/BCL-W inhibitor are not enough to determine the function of BCL-XL in specific human hematopoietic cell types. By using a genetic knock-down approach, we show here that BCL-XL is essential for human erythropoiesis and contributes to the survival and function of human HSPCs, multipotent progenitors (MPPs), and megakaryocytic progenitors. Our findings are only partly consistent with the murine data and clinical observations, and indicate a much broader and pronounced role of BCL-XL in human hematopoiesis than previously assumed.

## Materials and methods

### Lentiviruses

**Fig. 1:**
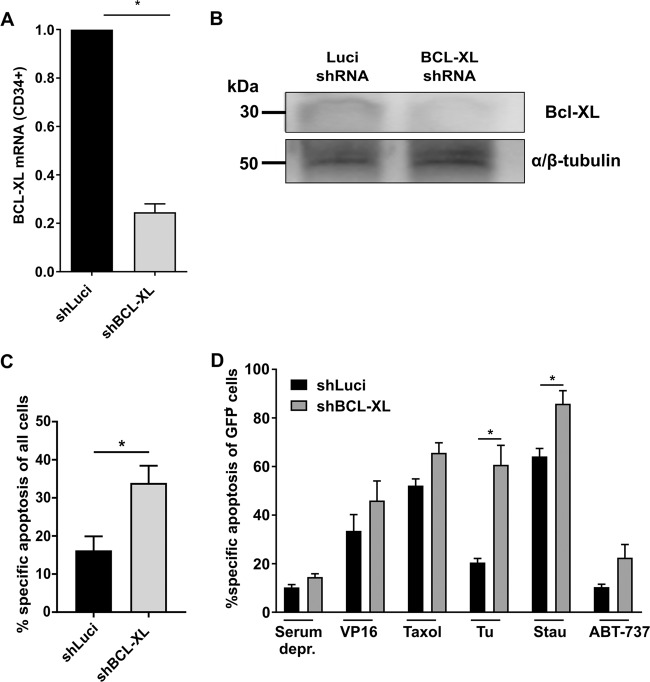
BCL-XL inhibition sensitizes human HSPCs to certain cytotoxic drugs. **a**, **b** Human CD34^+^ cells were transfected with lentiviral vectors targeting Luciferase (shLuci) or human BCL-XL (shBCL-XL). Knockdown efficiency of shBCL-XL was determined by qRT-PCR; mRNA expression was normalized to 18S. Bars represent mean ± SEM; *n* = 4 from four independent experiments; *p* = 0.0286 (**a**). In addition, protein levels were determined in CD34^+^GFP^+^ cells (**b**). **c** Apoptosis was determined in lentivirally transduced cells 24 h after end of viral transduction. **d** Lentivirally transduced CD34^+^ cells were subjected to cytotoxic treatment for 24 h; Control (Co, with serum), Serum deprivation (Serum depr.), 0.5 µg/ml VP16 (Etoposide), 0.05 µg/ml Taxol, 1 µg/ml Tunicamycin (Tu), 1 µM Staurosporine (Stau), 5 µM ABT-737. Cells were stained with Annexin V and 7-AAD and GFP^+^ cells were analyzed for percentage specific apoptosis by flow cytometry. Bars represent mean ± SEM, *n* = 4 from two independent experiments. The Mann–Whitney test was performed; *p* = 0.0286 for Tu and Stau.

pLeGOhU6 lentiviral vector with human U6 promoter and GFP or dTomato expression was used to generate shRNA expressing lentiviruses (Suppl. Table 1), while pLeGO-iG vector was used to overexpress BCL-2^23,24^. CD34^+^ cells were transduced with either one lentivirus (2x MOI 15, 24 h each) or two different lentiviruses (GFP and dTomato; 2x MOI 7.5 each). Knockdown efficiencies were determined on mRNA level (qRT-PCR; Fig. 1a and Supplementary Fig. 1a)^24^ and on protein level (western blot, antibody: Bcl-XL (54H6)mAb, Fig. 1b and Supplementary Fig. 1b) 24 h after completed transduction. All lentiviral experiments were conducted under biosafety level 2 (S2) conditions.

### Isolation and culture of human CD34^+^ cells

Umbilical cord blood was obtained immediately after birth from healthy women undergoing Cesarean deliveries after informed consent of parents and approval from the ethics committee of University Hospital Freiburg, Germany. CD34^+^ cells were isolated via MACS technology from mononuclear cells. Purity of cells was generally more than 90%. Cells were either used immediately after isolation or stored in liquid nitrogen using CS10 freezing medium (CryoStor) for later use. CD34^+^ cells were cultured at a density of 5 × 10^5^ − 1 × 10^6^ cells/ml in serum free StemPro-34 medium supplemented with ES-FBS, penicillin/streptomycin (Invitrogen) and human recombinant cytokines (Immunotools) including stem cell factor (SCF), FMS-like tyrosine kinase 3 ligand (FLT3L), thrombopoietin (TPO) (each 100 ng/ml) and interleukin-3 (IL-3; 20 ng/ml). Where indicated, the BCL-XL inhibitor A-1155463 (Selleckschem) was added. Lentivirally transduced cells were used for experiments 24 h after the transduction was completed.

### Apoptosis assay

Lentivirally transduced and/or untransduced CD34^+^ cells were treated with varying concentrations of cytotoxic drugs including etoposide (VP16), tunicamycin, taxol, staurosporine (Sigma-Aldrich) and ABT-737 (Selleck Chemicals). After 24 h, the cells were surface-stained with AnnexinV-Alexa fluor 647 (Biolegend) and 7-AAD (Sigma-Aldrich) and analyzed for apoptosis by flow cytometry. Percentage specific apoptosis was calculated as: 100 × (percentage living cells under control condition − percentage living cells under treatment)/percentage living cells under control condition. Control condition represented cell culture in the presence of ES-FBS along with cytokines. Percentage of GFP^+^ cells was analyzed to determine enrichment of transduced cells.

### Colony forming assays

Human CD34^+^ cells were seeded at a density of 1000 cells in MethoCult SF H4436 serum free medium. After 10 days of culture, various colony types were identified by light microscopy and total cell counts were determined. Percentages of HSPC, erythroid and myeloid cells were determined via flow cytometry. For investigating megakaryocytes, CD34^+^ cells were seeded in MegaCult-C Collagen based medium (containing 98% bovine collagen type I) with cytokines at a density of 5000 cells/1.5 ml. After 12 days of incubation, cells were harvested following digestion with collagenase type I (Stem Cell Technologies). Total cell counts were determined, and cells were analyzed by flow cytometry. Where indicated, the BCL-XL inhibitor A-1155463 (Selleckschem) was added.

### Human erythroid massive amplification assay (HEMA)

Transduced or untransduced CD34^+^ cells were cultured in a two-phase liquid culture assay to promote differentiation into erythroid cells^25^. Cells at a density of 2.5 × 10^5^/ml were cultured first for 10 days with IMDM supplemented with 20% FCS (Invitrogen), human SCF (10 ng/ml), β-estradiol (1 μM), dexamethasone (1uM) (Sigma-Aldrich), human IL-3 (1 ng/ml) (Immunotools) and erythropoietin (EPO) (1U/ml) (R&D Systems). After 10 days, medium was replaced with IMDM containing 20% FCS, human insulin (10 ng/ml) (Braun) and EPO (1U/ml), and cells were kept in culture for two more days. Medium was refreshed every four days and cells were counted. Cells were analyzed for various immature and mature erythroid populations by flow cytometry.

### Quantitative reverse transcription polymerase chain reaction

RNA was isolated (Quick RNA Micro Prep kit Zymo Research) and reversely transcribed to cDNA (Quantitec-Reverse transcription kit, QIAgen) as per manufacturer’s instructions. Real time Quantitative reverse transcription polymerase chain reaction (qRT-PCR) was performed by using BIO-RAD (CFX96 Touch) real time PCR detection system and SYBR Green master mix (Thermofisher). Expression of gene of interest was normalized to either 18S or 36B4 (Supplementary Table 1).

### Reverse transcriptase-multiplex ligation dependent probe amplification

RNA samples from erythroid cells isolated after HEMA culture were obtained by Fast Spin columns (Zymo Research). Reverse transcriptase-multiplex ligation dependent probe amplification (RT-MLPA) was performed as per manufacturer’s instructions (MRC Holland, R011-C1). Briefly, mRNA was reversely transcribed into cDNA, followed by its hybridization to two separate oligonucleotides (MLPA probes), one harboring a primer sequence and the other a stuffer sequence of specific length. The probes were then ligated and amplified during PCR. The resultant amplicons were of specific lengths and separated by capillary electrophoresis (ABI-3130xl Genetic Analyzer). Analysis was conducted using Sequence Pilot (JSI Medical Systems). Sum of all peaks was taken as 100%, and single peaks were normalized accordingly.

### Western blot

HEK293T cells were transfected with plasmids expressing Luci shRNA or BCL-XL shRNA and GFP^+^ cells were sorted. Additionally, lentivirally transduced human CD34^+^ were sorted for GFP^+^ cells. Proteins were purified and size fractioned by 12% SDS-PAGE under reducing conditions and transferred onto PVFD membranes by electroblotting. For detection of BCL-XL, anti–BCL-XL (54H6) rabbit mAb (Cell Signaling) was used. α/β-tubulin antibody (Cell Signaling) was used as a loading control. Secondary reagents were conjugated to peroxidase, and signal was detected by enhanced chemiluminescence.

### Xenotransplantation

All experiments were performed after approval from local ethics committee and in compliance with the German “Tierversuchsgesetz”. *Rag2*^*−/−*^*γc*^*−/−*^ mice were kept under specific pathogen-free conditions. 5-week-old juvenile mice were sub-lethally irradiated with 3Gy^24^. After 6 h, a progeny of 3 × 10^5^ transduced or untransduced human CD34^+^ cells were injected intravenously into the retrobulbar venous plexus under isofluorane anesthesia. Mice were sacrificed after 6–8 weeks, and single cell suspensions were harvested from bone marrow (BM) and spleens. Successful engraftment was defined as the presence of at least 0.5% human CD45^+^ cells in the murine BM^26^.

### Flow cytometry

Single cell suspensions obtained from colony forming assays or hematopoietic organs from mice were surface stained with monoclonal antibodies. Antibodies used for analysis of human immature stem and progenitor cells from colony forming assays: CD34 PE-Cy7(581), CD38 APC (HIT2), CD10 PE/PeCy5/APC (HI10a), CD45RA PerCP-Cy5.5 (HI100), CD90 APC-Cy7 (5E10) (Biolegend). Antibodies for erythroid cells from colony assays and HEMA: CD117 PE-Cy7 (104D2), CD71 APC/APC-Cy7 (CY1G4) (Biolegend), CD235a BV421 (HIR2) (BD biosciences). Antibodies for myeloid cells from colony forming assays: CD34 PE-Cy7 (581), CD33 PE/PerCP-Cy5.5 (WM53), CD14 APC (M5E2), CD115 BV421 (g-4D2-1E4), CD15 PeCy5 (W6D3), CD66b PerCP-Cy5.5 (G10F5) (Biolegend). Antibodies for megakaryocyte populations: CD41 PerCP-Cy5.5 (HIP8), CD61 APC (VI-PL2) (Biolegend). Antibodies for overall human and human myeloid cells in xenograft assay: CD45 Biotin (HI30), CD34 PE-Cy7/APC-Cy7 (581), CD33 PE/PerCP-Cy5.5 (WM53), CD15 PeCy5 (W6D3). Antibodies for human B cells in xenograft assay: CD19 PE-Cy7 (HIB19), IgM APC-Cy7 (MHM-88), CD10 APC (HI10a) (Biolegend). Antibody to detect murine cells in xenograft assay: CD45 PE-Cy7/PerCP-Cy5.5 (30-F11) (Biolegend). Streptavidin APC/V450 (Biolegend) were used as secondary antibodies. BD LSRFortessa was used for flow cytometry. Analyses were performed using FlowJo.

### Statistics

Statistical analyses were performed using the unpaired Mann–Whitney U test in GraphPad Prism 7 software. *P* values < 0.05 were considered statistically significant.

## Results

### Sustained BCL-XL knockdown has deleterious effects on human hematopoietic stem and progenitor cells

**Fig. 2:**
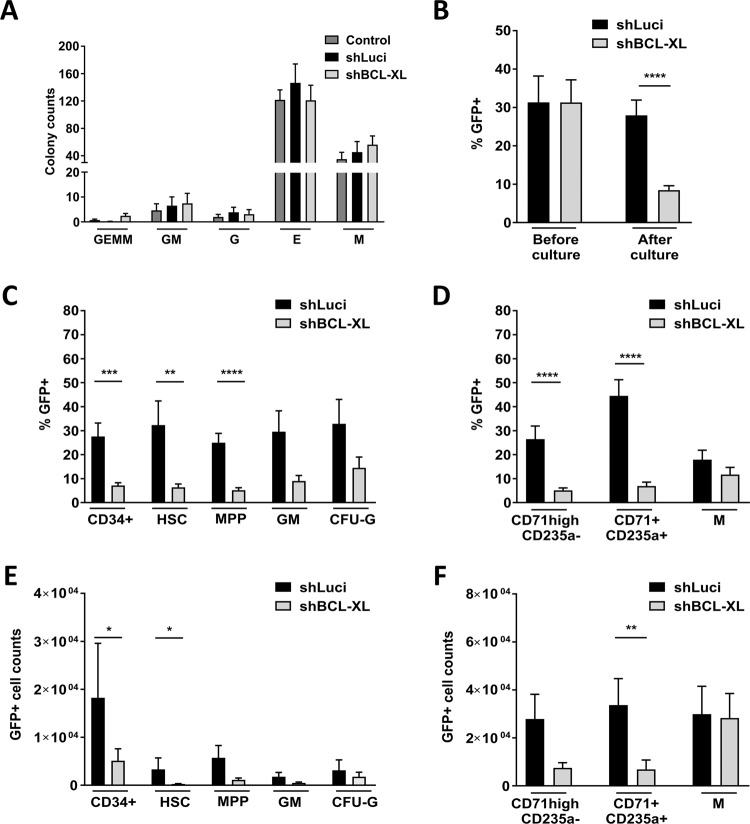
BCL-XL is important for the survival of differentiating CD34^+^ cells. **a**–**f** Human HSPC were seeded in MethoCult medium. After 10 days of culture, colonies were identified (granulocyte-erythrocyte-megakaryocyte-monocyte precursors (GEMM), granulocytic–monocytic precursors (GM), granulocytes (G), erythroid (E), and monocyte precursors (M)) and counted by light microscopy (**a**). GFP expression was determined before and after culture (**b**). Several hematopoietic populations were analyzed by flow cytometry along with GFP expression (**c**, **d**) and cell numbers (**e**, **f**). Supplementary Table 2 shows the immunophenotype of the hematopoietic populations. Abbreviations: hematopoietic stem cells (HSC), multipotent progenitors (MPP), granulocytic–monocytic progenitors (GM), Colony Forming Unit granulocytes (CFU-G). Bars represent mean ± SEM, *n* = 8–9 from five independent experiments. *p*-values: **b** ****p < 0.0001. **c** **p = 0.0025, ***p = 0.001, ****p < 0.0001. **d** ****p < 0.0001. **e** *p = 0.036 for CD34^+^ cells, *p = 0.0274 for HSC. **f** **p = 0.0037.

**Fig. 3:**
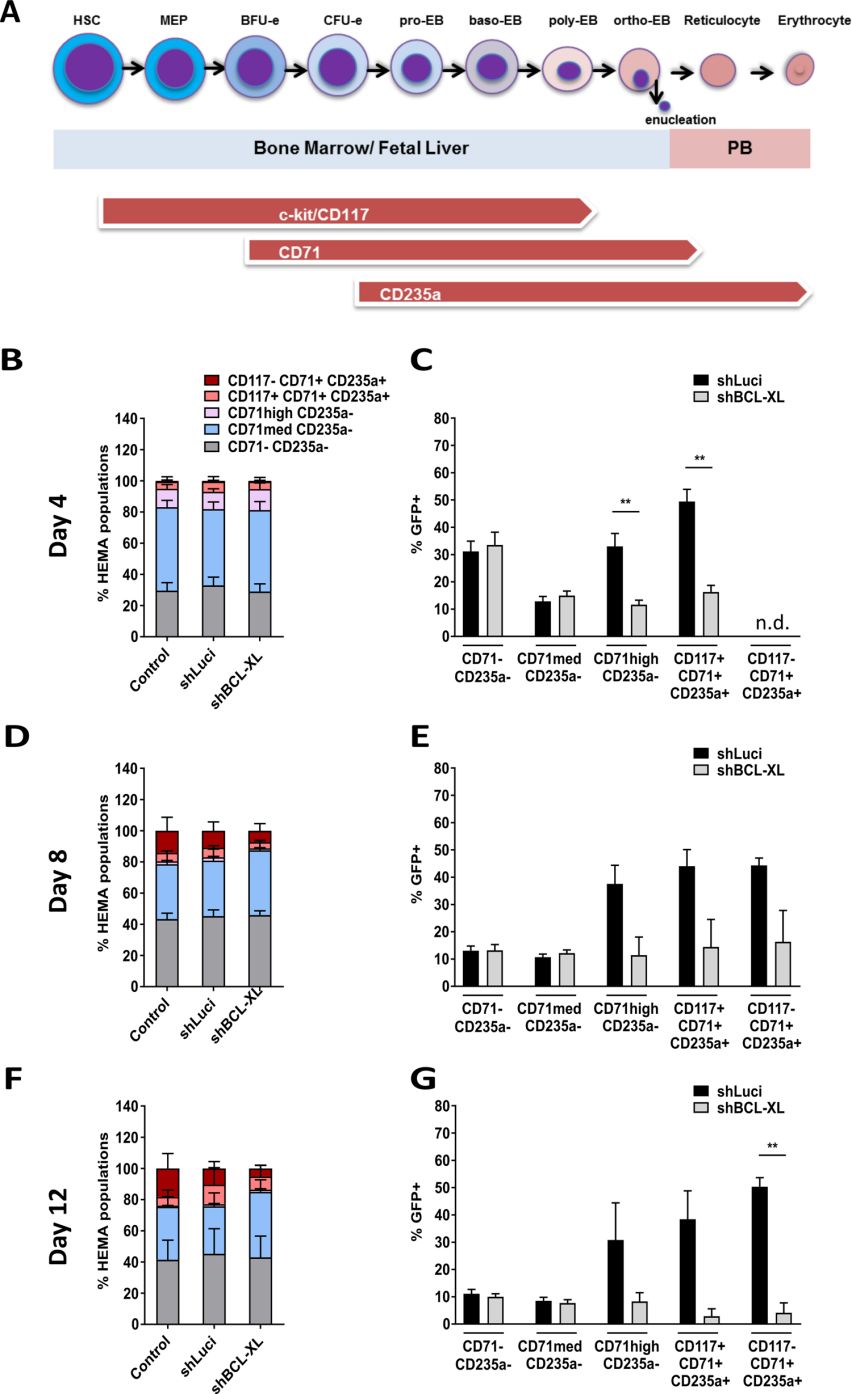
BCL-XL is essential for human erythropoiesis from very early stages. **a**–**f** Human HSPC were cultured in Human Erythroid Massive Amplification assay (HEMA) to study the impact of BCL-XL knockdown on different erythroid progenitors in vitro. Different populations were analyzed via flow cytometry at indicated time points of culture. Left panels show the stages of erythroid differentiation (**a**, **c**, **e**), the right panels show GFP^+^ cells in the individual subpopulations (**b**, **d**, **f**). Bars represent mean ± SEM, *n* = 5 from five independent experiments. *p*-values: **b**, **f** **p = 0.0079.

BH3-mimetics will be used mainly as part of combination therapies and side-effects on the HSPC pool will be influenced by the individual drug combination. Therefore, we first tested whether BCL-XL inhibition on its own or in combination with other cytotoxic drugs results in depletion of human CD34^+^ HSPCs. Knockdown efficiency of a BCL-XL specific shRNA was tested in HEK293T cells (Supp. Fig. 1a) and cord blood-derived CD34^+^ cells (Fig. 1a), which reduced BCL-XL mRNA levels to 11% and 25%, respectively. Knockdown was confirmed on protein level in both cell types (Fig. 1b, Suppl. Fig. 1b). Lentiviral BCL-XL knockdown on its own induced some apoptosis in CD34^+^ cells within 24 h of culture (Fig. 1c) and it increased sensitivity toward ER stress (tunicamycin) and pan-kinase inhibition (staurosporin). In contrast, BCL-XL knockdown in combination with serum deprivation, DNA damage (etoposide/VP16) or antimicrotubule assembly inhibition (taxol), did not increase susceptibility to apoptosis (Fig. 1c). To additionally inhibit BCL-2 and BCL-W in human HSPC, we combined BCL-XL knockdown with the BCL-2/BCL-XL/BCL-W inhibitor ABT-737 and observed a slight but not significant increase of apoptosis (Fig. 1c). We investigated how sustained BCL-XL inhibition affected the HSPC pool under more physiological conditions. Upon lentiviral transduction, GFP negative and positive cells were seeded in semi-solid methylcellulose medium containing cytokines driving hematopoietic differentiation. Colony numbers and types were determined after 10 days. Comparable differentiation and colony numbers were found among different groups (Fig. 2a). While transduction efficiencies and input of GFP^+^ cells were similar between the Luci and BCL-XL shRNA groups, significantly fewer GFP^+^ cells expressing BCL-XL shRNA were detected after culture indicating a selection disadvantage of these cells (Fig. 2b). Flow cytometric analysis of the subpopulations revealed that HSCs as well as MPPs were significantly depleted upon BCL-XL knockdown, while more mature granulocytic and monocytic cells were less dependent on BCL-XL expression (Fig. 2c–f, Supplemnetary Figs. 2 and 3, Supplementary Table 2). We also detected significantly fewer GFP^+^ erythroid cells when BCL-XL was downregulated (Fig. 2d, f). This is in line with the expression data of human hematopoietic cells, which show strong BCL-XL mRNA upregulation in erythroid cells, and indicates a fundamental role of BCL-XL during human erythropoiesis^27^.

### BCL-XL is essential for human erythropoiesis

To specifically analyze erythroid differentiation of human CD34^+^ cells, we applied the HEMA protocol, which sequentially induces expansion and erythroid differentiation of human HSPC^25^. By using cell surface markers for the transferrin receptor (CD71), glycophorin A (CD235a) and c-Kit (CD117), we could detect different maturation stages from the earliest cells committed to the erythroid lineage (BFU-e; CD117^+^CD71^med^ CD235a^−^) to orthochromatic proerythroblasts and reticulocytes (CD117^−^CD71^high^CD235a^+^) (Supplemnetary Table 3, Fig. 3a, Supplementary Fig. 4). Flow cytometric analysis was performed after 4, 8, and 12 days and showed a gradual increase of more differentiated cell types over time in controls (Fig. 3b–g, left panels, Supplementary Fig. 5). Analysis of the Luci shRNA expressing controls revealed that the frequency of GFP^+^ cells decreased over time in non-erythroid cells while it remained stable in erythroid cells. This indicates that virally infected HSPCs are skewed towards erythroid differentiation or that, alternatively, progenitors committed to the erythroid lineage are preferentially infected by lentiviruses. In contrast, repression of BCL-XL severely impacted survival of immature erythroid cells (CD117^+^CD71^high^CD235a^−^) from day 4 of culture. The effect was transmitted to late stages of erythroid maturation in the following days (Fig. 3, right panels, Supplementary Fig. 5). Together, these data show that BCL-XL expression is crucial for generation and maintenance of the human erythroid system and “BCL-XL addiction” already starts in the early-stage cells committed to the erythroid lineage.

In addition, we performed MegaCult experiments to study megakaryocytic development in vitro. Cells differentiated to the stage of CD61^+^CD41^+^ were considered as megakaryocytes. BCL-XL knockdown negatively impacted their survival, albeit not significantly (Supplementary Fig. 6).

### The BCL-XL inhibitor A-1155463 depletes human erythroid cells and, after prolonged treatment, immature stem, and progenitor cells

**Fig. 4:**
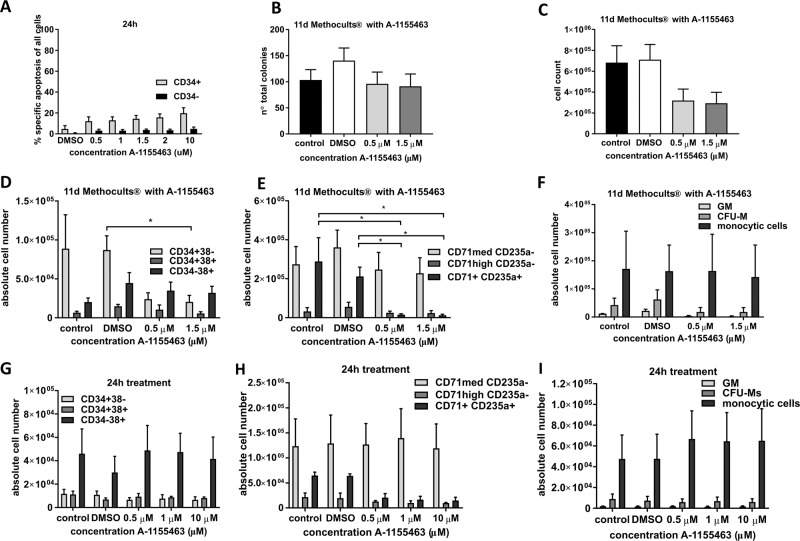
Treatment with A-1155463 confirms the detrimental effects of BCL-XL inhibition on erythroid cells and CD34^+^ 38^−^ stem and progenitor cells. **a** Freshly isolated CD34^+^ and CD34^−^ cells were treated with the indicated concentrations of the BCL-XL inhibitor A-1155463. After 24 h apoptosis was measured by flow cytometry and percentage specific apoptosis was determined (*n* = 4–5). **b**–**f** Human HSPC were differentiated in Methocult® culture in the presence of 0.5 and 1.5 µM of the inhibitor. After 11 days, total colony numbers (**b**) as well as total cell numbers (**c**) were determined (*n* = 4) and immature (**d**), erythroid (**e**), and myeloid cell populations (**f**) were analyzed by flow cytometry. **g**–**i** CD34^+^ cells were differentiated in the absence of the inhibitor for 11 days, and their progenies were isolated and treated with A-1155463 for 24 h. Immature (**g**), erythroid (**h**), and myeloid cell populations (**i**) were analyzed by flow cytometry. Abbreviations: granulocytic–monocytic progenitors (GM), colony forming unit monocytes (CFU-M). Bars represent mean ± SEM, *n* = 2–3. p-values: (**d**, **e**) *p = 0.0286.

To confirm our findings, we used the potent and selective BCL-XL inhibitor A-1155463^28^. We first treated immature CD34^+^ and mature CD34^−^ cells freshly isolated from cord blood with increasing doses of the inhibitor. Specific apoptosis determined 24 h later was higher in CD34^+^ cells but consistently <20%, even when high concentrations were used (Fig. 4a). We added the inhibitor to the Methocult medium and analyzed the emerging colonies 11 days later. Colony numbers were not reduced (Fig. 4b) but total cell count was lower when BCL-XL was inhibited (Fig. 4c). This indicates that HSPCs were not depleted within the time frame required for colony formation but that emerging colonies were smaller. Further analysis revealed that immature CD34^+^38^−^ HSPCs were depleted within these 11 days. Similarly, CD71^+^CD235^+^ erythroid cells were significantly reduced while all other cell types analyzed were present in normal numbers (Fig. 4d–f).

So far, all experiments were performed on immature or differentiating cells. To analyze the effects of BCL-XL inhibition on already differentiated cells, we cultured CD34^+^ cells in Methocult medium and isolated their progenies after 11 days. Differentiated cells were treated with A-1155463 for 24 h and analyzed by flow cytometry. Mature erythroid cells were depleted completely while neither myeloid cells nor immature stem and progenitor cells were affected (Fig. 4g–i). In sum, both our RNAi and inhibitor data revealed that erythroid cells are very sensitive to BCL-XL inhibition. In addition, BCL-XL inhibition by shRNA or treatment with A-1155463 led to depletion of immature stem and progenitor cells, especially when the inhibition was sustained.

### BCL-XL knockdown impedes engraftment of human HSPCs during xenotransplantation

**Fig. 5:**
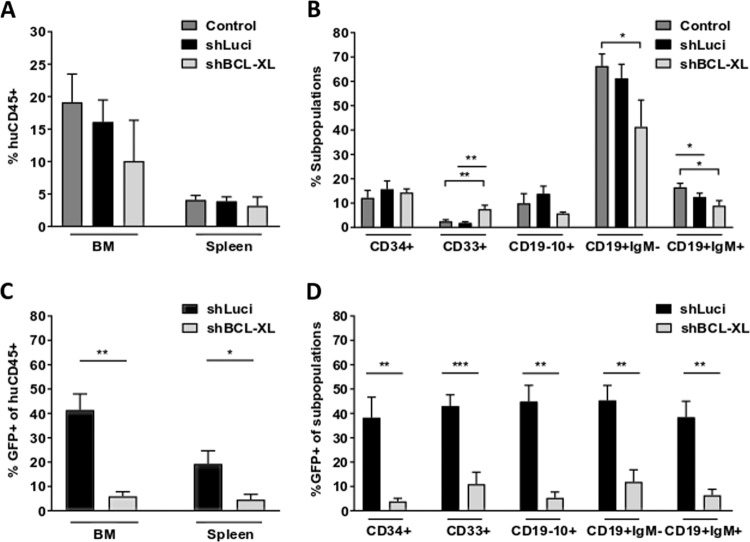
Human cells lacking BCL-XL show poor engraftment in xenograft mice. **a**–**d** Lentivirally transduced or untransduced human HSPC were transplanted in *Rag2*^*−/−*^*γc*^*−/−*^ mice after sub-lethal irradiation. Mice were sacrificed 7–8 weeks after transplantation and bone marrow (BM) and spleen were analyzed for the presence of various human hematopoietic populations by flow cytometry (**a**, **b**) as well as for GFP expression in the individual subpopulations (**c**, **d**). Bars represent mean ± SEM, *n* = 6-10 from six independent experiments. *p*-values: **b** **p = 0.0047 for control vs shBCL-XL CD33^+^ cells; **p = 0.003 for shLuci vs shBCL-XL CD33^+^ cells; *p = 0.035 control vs shBCL-XL CD19^+^IgM^−^ cells; *p = 0.0431 for control vs shLuci CD19^+^IgM^+^ cells; *p = 0.014 for control vs shBCL-XL CD19^+^IgM^+^ cells (**c**) **p = 0.0017, *p = 0.0312 (**d**) **p = 0.0017 for CD34^+^ and CD19^−^10^+^ cells; ***p = 0.001; **p = 0.003 for CD19^+^IgM^−^, and CD19^+^IgM^+^ cells.

To test whether the detrimental effects of BCL-XL knockdown on HSCs in vitro impact their reconstitution potential in vivo, xenotransplantation experiments were performed. Lentivirally transduced CD34^+^ cells were injected intravenously in 5-week old *Rag2*^*−/−*^*γc*^*−/−*^ mice subjected to sub-lethal irradiation. Eight weeks later, bone marrow and spleens of recipient mice were analyzed for human engraftment, differentiation and GFP expression (Supplementary Fig. 7). While comparable human engraftment and myeloid/lymphatic differentiation were observed in all groups (Fig. 5a, b), significant differences were observed in the engraftment of shRNA expressing GFP^+^ cells. Luci-shRNA expressing CD34^+^ cells gave rise to 41.35% and 19.24% human cells in BM and spleen, respectively. In contrast, significantly lower GFP^+^ cell percentages were detected in both organs (5.91% and 4.57% in BM and spleen, respectively) when BCL-XL was knocked down (Fig. 5c). Both myeloid and lymphatic subpopulations as well as immature CD34^+^ cells were affected in a similar manner indicating that loss of BCL-XL expression resulted in a defect of stem cells or early progenitors (Fig. 5d). Analysis of human erythroid cells was hampered by their lack of CD45^+^ expression and the low degree of specificity of other antibodies for detection of erythroid cells. Nevertheless, normal erythroid differentiation in vivo could not be expected based on the severe phenotype in vitro.

### BCL-2 overexpression but not BIM inhibition rescues human stem and erythroid cells

**Fig. 6:**
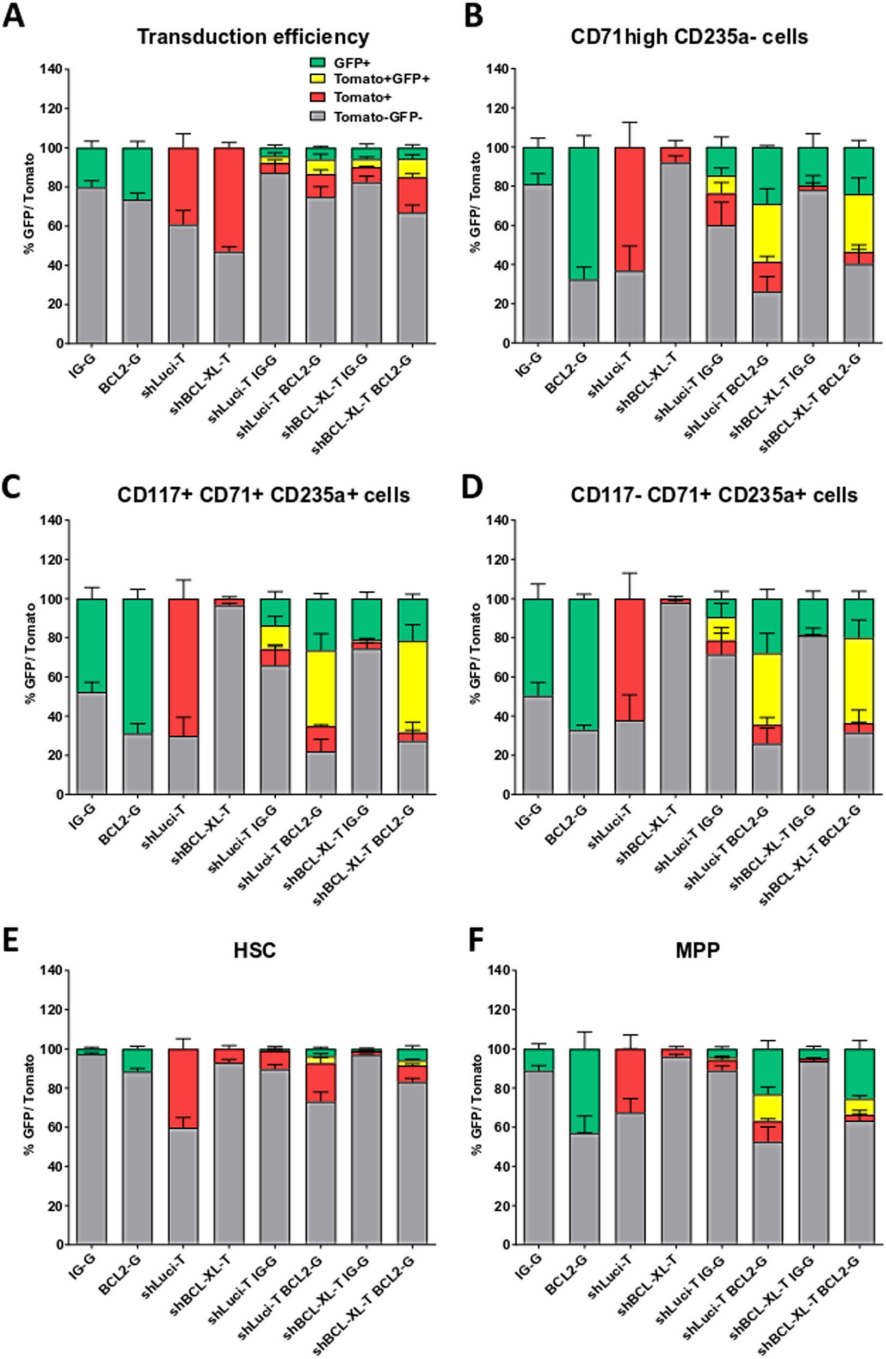
BCL-2 overexpression reverses all effects of BCL-XL deficiency. **a**–**f** Human CD34^+^ HSPC were lentivirally transduced with one or two lentiviruses as indicated; pLeGO-iG empty vector with GFP (IG-G); pLeGO-iG BCL-2 overexpressing vector with GFP (BCL2-G); pLeGOhU6 with shLuci and dTomato (shLuci-T); pLeGOhU6 with shBCL-XL and dTomato (shBCL-XL-T); pLeGOhU6 with shLuci and dTomato + pLeGO-iG empty vector with GFP (shLuci-T IG-G); pLeGOhU6 with shLuci and dTomato + pLeGO-iG BCL-2 overexpressing vector with GFP (shLuci-T BCL2-G); pLeGOhU6 with shBCL-XL and dTomato + pLeGO-iG empty vector with GFP (shBCL-XL-T IG-G); pLeGOhU6 with shBCL-XL and dTomato + pLeGO-iG BCL-2 overexpressing vector with GFP (shBCL-XL-T BCL2-G) (**a**) and cultured for 8 days under HEMA culture conditions (**b**–**d**) or in MethoCult plates (**e**, **f**). Bars represent percentage GFP^+^, percentage Tomato^+^, percentage double-negative, and percentage double-positive cells (mean ± SEM), *n* = 4–8 from four independent experiments.

The anti-apoptotic function of BCL-XL is based on its inhibitory effect on pro-apoptotic signals, particularly on BH3-only proteins and BAX/BAK. Inhibition of detrimental signals or pro-apoptotic BCL-2 proteins, therefore, should rescue the deleterious effects of BCL-XL loss. Correspondingly, overexpression of BCL-2 should be able to compensate for loss of BCL-XL due to the overlapping functions of both anti-apoptotic proteins. We therefore overexpressed BCL-2 in CD34^+^ cells with BCL-XL knockdown. To distinguish between the two lentiviral systems, we replaced GFP with dTomato in the BCL-XL and Luci shRNA vectors, while retaining GFP expression in the BCL-2 overexpressing virus. The transduction efficiency is shown in Fig. 6a. As expected, BCL-2 overexpression fully compensated for BCL-XL loss. All BCL-XL depleted immature erythroid cells generated in the HEMA assay survived efficiently when BCL-2 was overexpressed (Fig. 6b–d, Supplementary Fig. 8). Similar protective effects could be observed in HSCs and MPPs generated in colony forming assays, although the yield of lentivirally transduced cells was lower than in HEMA assays (Fig. 6e, f).

**Fig. 7:**
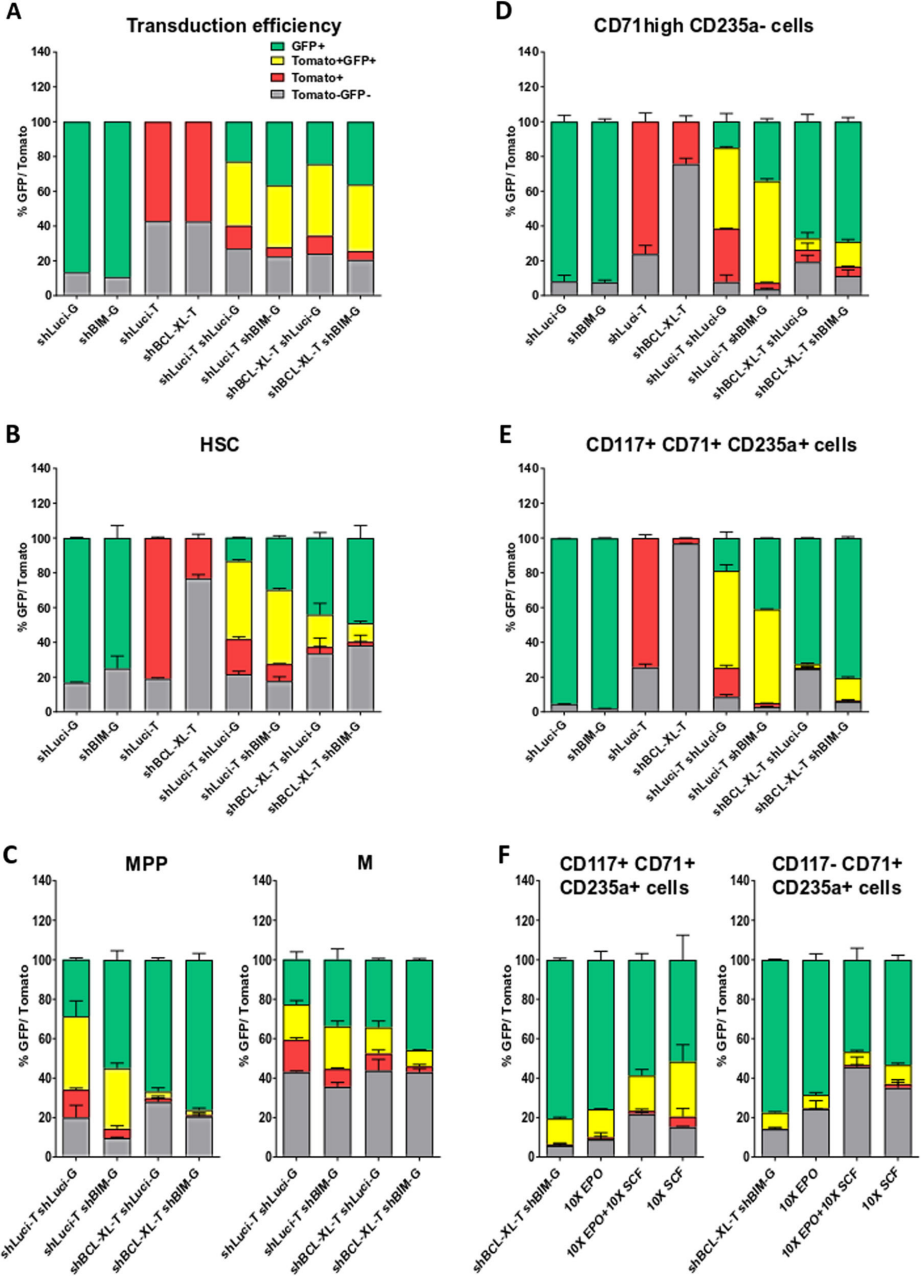
BIM knockdown does not rescue BCL-XL deficient erythroid cells. **a**–**f** Human CD34^+^ HSPC were lentivirally transduced as indicated; pLeGOhU6 with shLuci and GFP (shLuci-G); pLeGOhU6 with shBIM and GFP (shBIM-G); pLeGOhU6 with shLuci and dTomato (shLuci-T); pLeGOhU6 with shBCL-XL and dTomato (shBCL-XL-T); pLeGOhU6 with shLuci and dTomato + pLeGOhU6 with shLuci and GFP (shLuci-T shLuci-G); pLeGOhU6 with shLuci and dTomato + pLeGOhU6 with shBIM and GFP (shLuci-T shBIM-G); pLeGOhU6 with shBCL-XL and dTomato + pLeGOhU6 with shLuci and GFP (shBCL-XL-T shLuci-G); pLeGOhU6 with shBCL-XL and dTomato + pLeGOhU6 with shBIM and GFP (shBCL-XL-T shBIM-G), and transduction efficiency was determined after 48 h, *n* = 1 (CD34^+^ cells from two cord bloods) (**a**). MethoCult experiments (**b**, **c**) as well as an 8-day long HEMA culture (**d**, **e**) were performed (*n* = 2 from one independent experiment). In addition, erythroid cells transduced with shBCL-XL (dTomato) and shBIM (GFP) were cultured for 8 days with 10-fold concentrations of EPO and/or SCF (**f**) CD117^+^CD71^+^CD235a^+^ and CD117^−^CD71^+^CD235a^+^ cells.

The BH3-only protein BIM was described to be the most important BH3-only protein for the murine hematopoietic system and its loss led to pathological survival of HSPC, lymphatic and myeloid cells^24,29,30^. We expected that at least, part of apoptosis induced by BCL-XL knock down could be rescued by additional loss of BIM. While transduction was very efficient (Fig. 7a), we did not observe increased survival of cells expressing BCL-XL shRNA-dTomato together with BIM shRNA-GFP, neither in HEMA nor in colony forming assays (Fig. 7b–e, Supplmentary Fig. 9). This demonstrated that simultaneous downregulation of BIM was not sufficient to rescue BCL-XL depleted erythroid cells and HSPCs. Survival of erythroid progenitor cells is fostered by the cytokines EPO and SCF, which were shown to increase expression of BCL-XL while suppressing several BH3-only proteins^31,32^. To tip the balance further in favor of survival signals, we combined BIM knock-down with increased cytokine stimulation by using ten-fold concentrations of EPO and/or SCF. Again, no increased survival of erythroid cells lacking BCL-XL could be achieved (Fig. 7f, Supplementary Fig. 9e, f).

We showed earlier that most transplantation-associated apoptosis is induced by BIM, in both murine systems and human xenograft models^24^. Despite the negative results in the in vitro experiments, we xenotransplanted human CD34^+^ cells co-transduced with BCL-XL shRNA-dTomato and BIM shRNA-GFP into sub-lethally irradiated *Rag2*^*−/−*^*γc*^*−/−*^ mice. Six weeks after transplantation, we were not able to harvest any dTomato^+^GFP^+^ (Tom^+^GFP^+^) cells (Supplmentary Fig. 10).

In sum, while BCL-XL deficiency could be compensated by BCL-2 overexpression, loss of BIM did not mitigate its detrimental effects. We hypothesized that other BH3-only proteins or BCL-XL interaction partners are responsible for the programmed death of the various hematological cell types. To identify potential candidates for erythroid progenitors, we performed expression analysis on cells cultured for 12 days under HEMA conditions. We identified PUMA and BNIP3L (NIX) as potential candidates responsible for erythroid cell apoptosis (Supplementary Fig. 11).

## Discussion

Hematological side effects are the major cause of treatment related morbidity and mortality in oncology, and they can limit therapy efficacy by requiring treatment delays and dose reduction. Because of the severe thrombocytopenia, the BH3 mimetic Navitoclax was even declined FDA approval despite showing convincing anticancer effects. On the contrary, the BCL-2 specific inhibitor Venetoclax was approved by FDA for CLL and small lymphocytic leukemia, indicating that BCL-2 inhibition is better tolerated than combined BCL-2/BCL-XL/BCL-w inhibition. Nevertheless, it can be expected that exclusive BCL-2 inhibitors will be of very limited use for most malignancies, and that not only MCL-1 inhibitors but also novel and specific BCL-XL inhibitors will find their way into clinic trials.

Here we provide a detailed in vitro and in vivo description of how efficient and specific BCL-XL inhibition affects the human hematopoietic system. The most detrimental effects were observed within the erythroid lineage. While the earliest committed progenitors developed normally, late BFU-e/CFU-e were almost completely depleted in the absence of BCL-XL. This stands in contrast to murine erythropoiesis, where the role of BCL-XL is restricted to reticulocytes and mature erythrocytes^14,15,20^. Why is BCL-XL so important in erythropoiesis? One answer can be found in its regulation by EPO and SCF. Erythropoiesis must promptly fulfill demands of oxygen supply under stress conditions, such as hypoxia caused by bleedings, high altitude or hemolysis, and thus is a highly plastic system. Especially, progenitor cells (up to the stage of basophilic erythroblasts) have a high proliferative capacity. EPO and SCF are the major pro-survival and proliferative signals for these early erythroid progenitors and their absence or presence defines whether a few progenitors differentiate into red blood cells or they increase proliferation^33–36^. BCL-XL is the main pro-survival target downstream of EPO^37–39^. At the same time, EPO promotes survival by downregulating *BIM* mRNA expression and inducing BIM protein degradation in an ERK-dependent manner^40,41^. During terminal differentiation, BCL-XL is further upregulated in murine and human erythroblasts while BCL-2 and MCL-1 are not expressed in these cells^27^. Erythroblasts produce copious amounts of hemoglobin and it is conceivable that BCL-XL is required to counteract ER stress and oxidative damage in these cells. We expected to rescue early, cytokine-responsive erythroid progenitors by suppressing BIM expression but observed no increased survival of cells expressing both BCL-XL and BIM shRNA. To suppress all pro-apoptotic factors downstream of cytokine withdrawal, we increased EPO and/or SCF concentrations but were again not able to keep BCL-XL deficient erythroid progenitors alive. In contrast, BCL-2 overexpression was able to reverse all phenotypes caused by BCL-XL knockdown implicating that BH3-only proteins other than BIM contribute to programmed death in the erythroid lineage. Prime candidates are PUMA and NOXA, which are known to mediate ER stress-induced apoptosis in many cell types^42–44^ and have been implicated in stress erythropoiesis, respectively^45^. A recent mouse model showed that indeed, loss of PUMA can alleviate anemia caused by BCL-XL deficiency^15^, and our expression studies revealed strong *PUMA* upregulation during human erythroid differentiation. In addition, BCL-XL might be required to keep BNIP3L (NIX) in check. This “atypical” BH3-only protein is upregulated during terminal differentiation and required for organelle autophagy (i.e. mitophagy), but its unleashed activation can induce apoptosis^46–48^.

Since the best described side effect of clinical BCL-XL inhibition is thrombocytopenia^21^, we investigated the knockdown effect on megakaryopoiesis. Based on the observation that thrombocytopenia was transient in most patients and counteracted by increased megakaryopoiesis it was hypothesized that BCL-XL inhibition only leads to demise of platelets while not affecting their production. We now show that, like in the mouse hematopoietic system, also megakaryocyte development is negatively affected by BCL-XL inhibition indicating that continuous BCL-XL inhibition might severely affect platelet production over time.

The most unexpected finding of our study was the partial depletion of HSCs and MPPs in the absence of BCL-XL in vitro. Accordingly, BCL-XL deficient CD34^+^ cells showed very poor engraftment in xenograft mice. This could not be foreseen from mouse models where fetal liver-derived HSCs were able to give rise to all hematological lineages and maintain hematopoiesis in a life-long manner, except mature erythrocytes^13,14^. Similarly, BCL-XL deletion in adult mice did not affect the HSPC compartment^15^. Our finding indicates a broader role of BCL-XL for human than for murine hematopoiesis. Since loss of human HSPC was not complete, it is very likely that other anti-apoptotic BCL-2 proteins, especially MCL-1, have an important role in HSPC survival^5,49^. Regarding the in vivo data, it should be kept in mind that xenograft models are of only limited feasibility in testing function and stress resistance of human stem cells^50^. Xenotransplantation comes along with incompatibility between human HSCs and the murine microenvironment, thereby imposing very high levels of stress on the transplanted cells. Under such conditions, endogenous BCL-XL might be required to counteract BH3-only proteins exclusively engaged in the imperfect microenvironment but not during clinical hematopoietic stem cell transplantation (HSCT). We found earlier that both transplantation and xenotransplantation-associated apoptosis are mediated primarily by the BH3-only proteins BIM and BMF^24^. Even their transient inhibition during the period of transplantation and early engraftment resulted in better transplantation outcomes^51,52^. We, therefore, postulated that additional inhibition of BIM would at least partially improve human engraftment during xenotransplantation but, unexpectedly, did not observe any beneficial effects of this manipulation. In sum, we provide evidence that BCL-XL plays an important role in human HSCs and MPPs, and that its continuous and efficient inhibition could severely affect hematopoiesis. Despite the limitations of our experimental approach, we would strongly advise not to use BCL-XL inhibitors around the time of HSCT to guarantee successful engraftment of donor cells. Additionally, we expect more severe hematological side effects, particularly thrombocytopenia and anemia once more efficient BCL-XL inhibitors find their way into clinics.

Finally, we propose to test novel BCL-XL inhibitors in preclinical and clinical trials for selected hematopoietic malignancies based on the data presented here. Candidate disorders for BCL-XL inhibition could be polycythemia vera, the very rare acute erythroid leukemia (AML M6), essential thrombocytosis and acute megakaryocytic leukemia (AML M7), and perhaps even minimally differentiated acute myeloblastic leukemia (AML M0). Along this line, BCL-XL was shown to be overexpressed in polycythemia vera^53^. Pre-clinical studies will be required to determine safe therapeutic window for the administration of specific and efficient BCL-XL inhibitors.

## Supplementary information

Legends for Suppl. material

Suppl. Table 1

Suppl. Table 2

Suppl. Table 3

Suppl. Figure 1

Suppl. Figure 2

Suppl. Figure 3

Supp. Figure 4

Suppl. Figure 5

Suppl. Figure 6

Suppl. Figure 7

Suppl. Figure 8

Suppl. Figure 9

Suppl. Figure 10

Suppl. Figure 11

merged suppl. data

## References

[1] Kollek M, Muller A, Egle A, Erlacher M (2016). Bcl-2 proteins in development, health, and disease of the hematopoietic system. FEBS J..

[2] Adams JM, Cory S (2018). The BCL-2 arbiters of apoptosis and their growing role as cancer targets. Cell Death Differ..

[3] Veis DJ, Sorenson CM, Shutter JR, Korsmeyer SJ (1993). Bcl-2-deficient mice demonstrate fulminant lymphoid apoptosis, polycystic kidneys, and hypopigmented hair. Cell.

[4] Rinkenberger JL, Horning S, Klocke B, Roth K, Korsmeyer SJ (2000). Mcl-1 deficiency results in peri-implantation embryonic lethality. Genes Dev..

[5] Opferman JT (2005). Obligate role of anti-apoptotic MCL-1 in the survival of hematopoietic stem cells. Science.

[6] Merino D (2018). BH3-mimetic drugs: blazing the trail for new cancer medicines. Cancer cell.

[7] Kipps TJ (2015). A phase 2 study of the BH3 mimetic BCL2 inhibitor navitoclax (ABT-263) with or without rituximab, in previously untreated B-cell chronic lymphocytic leukemia. Leuk. lymphoma.

[8] Roberts AW (2016). Targeting BCL2 with venetoclax in relapsed chronic lymphocytic leukemia. N. Engl. J. Med..

[9] Chang BS (1999). The BH3 domain of Bcl-x(S) is required for inhibition of the antiapoptotic function of Bcl-x(L). Mol. Cell. Biol..

[10] Boise LH (1993). bcl-x, a bcl-2-related gene that functions as a dominant regulator of apoptotic cell death. Cell..

[11] Vela L, Gonzalo O, Naval J, Marzo I (2013). Direct interaction of Bax and Bak proteins with Bcl-2 homology domain 3 (BH3)-only proteins in living cells revealed by fluorescence complementation. J. Biol. Chem..

[12] Edlich F (2011). Bcl-x(L) retrotranslocates Bax from the mitochondria into the cytosol. Cell.

[13] Motoyama N (1995). Massive cell death of immature hematopoietic cells and neurons in Bcl-x-deficient mice. Science.

[14] Motoyama N, Kimura T, Takahashi T, Watanabe T, Nakano T (1999). bcl-x prevents apoptotic cell death of both primitive and definitive erythrocytes at the end of maturation. J. Exp. Med..

[15] Delbridge AR (2017). The BH3-only proteins BIM and PUMA are not critical for the reticulocyte apoptosis caused by loss of the pro-survival protein BCL-XL. Cell Death Dis..

[16] Chang J (2016). Clearance of senescent cells by ABT263 rejuvenates aged hematopoietic stem cells in mice. Nat. Med..

[17] Josefsson EC (2011). Megakaryocytes possess a functional intrinsic apoptosis pathway that must be restrained to survive and produce platelets. J. Exp. Med..

[18] Dolznig H (2002). Apoptosis protection by the Epo target Bcl-X(L) allows factor-independent differentiation of primary erythroblasts. Curr. Biol.: CB.

[19] Shearn AI (2012). Bcl-x inactivation in macrophages accelerates progression of advanced atherosclerotic lesions in Apoe(−/−) mice. Arteriosclerosis, thrombosis, Vasc. Biol..

[20] Wagner KU (2000). Conditional deletion of the Bcl-x gene from erythroid cells results in hemolytic anemia and profound splenomegaly. Development.

[21] Mason KD (2007). Programmed anuclear cell death delimits platelet life span. Cell.

[22] Roberts AW (2012). Substantial susceptibility of chronic lymphocytic leukemia to BCL2 inhibition: results of a phase I study of navitoclax in patients with relapsed or refractory disease. J. Clin. Oncol. : Off. J. Am. Soc. Clin. Oncol..

[23] Roelz R, Pilz IH, Mutschler M, Pahl HL (2010). Of mice and men: human RNA polymerase III promoter U6 is more efficient than its murine homologue for shRNA expression from a lentiviral vector in both human and murine progenitor cells. Exp. Hematol..

[24] Labi V (2013). Haematopoietic stem cell survival and transplantation efficacy is limited by the BH3-only proteins Bim and Bmf. EMBO Mol. Med..

[25] Migliaccio G (2010). Humanized culture medium for clinical expansion of human erythroblasts. Cell Transplant..

[26] Krombholz CF (2016). Long-term serial xenotransplantation of juvenile myelomonocytic leukemia recapitulates human disease in Rag2−/−gammac−/− mice. Haematologica.

[27] Gregoli PA, Bondurant MC (1997). The roles of Bcl-X(L) and apopain in the control of erythropoiesis by erythropoietin. Blood.

[28] Tao ZF (2014). Discovery of a potent and selective BCL-XL inhibitor with in vivo activity. ACS medicinal Chem. Lett..

[29] Bouillet P (1999). Proapoptotic Bcl-2 relative Bim required for certain apoptotic responses, leukocyte homeostasis, and to preclude autoimmunity. Science.

[30] Shinjyo T (2001). Downregulation of Bim, a proapoptotic relative of Bcl-2, is a pivotal step in cytokine-initiated survival signaling in murine hematopoietic progenitors. Mol. Cell. Biol..

[31] Koulnis M, Porpiglia E, Hidalgo D, Socolovsky M (2014). Erythropoiesis: from molecular pathways to system properties. Adv. Exp. Med. Biol..

[32] Zeuner A (2003). Stem cell factor protects erythroid precursor cells from chemotherapeutic agents via up-regulation of BCL-2 family proteins. Blood.

[33] Vannucchi Alessandro Maria, Bianchi Lucia, Cellai Cristina, Paoletti Francesco, Carrai Valentina, Calzolari Anna, Centurione Lucia, Lorenzini Rodolfo, Carta Claudio, Alfani Elena, Sanchez Massimo, Migliaccio Giovanni, Migliaccio Anna Rita (2001). Accentuated response to phenylhydrazine and erythropoietin in mice genetically impaired for their GATA-1 expression (GATA-1low mice). Blood.

[34] Jelkmann W (2004). Molecular biology of erythropoietin. Intern. Med..

[35] Testa U (1996). Expression of growth factor receptors in unilineage differentiation culture of purified hematopoietic progenitors. Blood.

[36] Testa U (2004). Apoptotic mechanisms in the control of erythropoiesis. Leukemia.

[37] Silva M (1996). Erythropoietin can promote erythroid progenitor survival by repressing apoptosis through Bcl-XL and Bcl-2. Blood.

[38] Socolovsky M, Fallon AE, Wang S, Brugnara C, Lodish HF (1999). Fetal anemia and apoptosis of red cell progenitors in Stat5a−/−5b−/− mice: a direct role for Stat5 in Bcl-X(L) induction. Cell.

[39] Aispuru GR (2008). Erythroid expansion and survival in response to acute anemia stress: the role of EPO receptor, GATA-1, Bcl-xL and caspase-3. Cell Biol. Int..

[40] Koulnis M (2012). Contrasting dynamic responses in vivo of the Bcl-xL and Bim erythropoietic survival pathways. Blood.

[41] Abutin RM (2009). Erythropoietin-induced phosphorylation/degradation of BIM contributes to survival of erythroid cells. Exp. Hematol..

[42] Tan S (2014). PUMA mediates ER stress-induced apoptosis in portal hypertensive gastropathy. Cell Death Dis..

[43] Wali JA (2014). The proapoptotic BH3-only proteins Bim and Puma are downstream of endoplasmic reticulum and mitochondrial oxidative stress in pancreatic islets in response to glucotoxicity. Cell Death Dis..

[44] Pihan P, Carreras-Sureda A, Hetz C (2017). BCL-2 family: integrating stress responses at the ER to control cell demise. Cell Death Differ..

[45] Wensveen FM (2013). BH3-only protein Noxa contributes to apoptotic control of stress-erythropoiesis. Apoptosis.

[46] Aerbajinai W, Giattina M, Lee YT, Raffeld M, Miller JL (2003). The proapoptotic factor Nix is coexpressed with Bcl-xL during terminal erythroid differentiation. Blood.

[47] Diwan A (2007). Unrestrained erythroblast development in Nix-/- mice reveals a mechanism for apoptotic modulation of erythropoiesis. Proc. Natl Acad. Sci. USA.

[48] Sandoval H (2008). Essential role for Nix in autophagic maturation of erythroid cells. Nature.

[49] Vo TT (2012). Relative mitochondrial priming of myeloblasts and normal HSCs determines chemotherapeutic success in AML. Cell.

[50] Demmerath, E. M., Bohler, S., Kunze, M. & Erlacher, M. In vitro and in vivo evaluation of possible pro-survival activities of PGE2, EGF, TPO and FLT3L on human hematopoiesis. *Haematologica* **104**, 669–677 (2018). 10.3324/haematol.2018.191569 PMC6442978 30442724

[51] Kollek M (2017). Transient apoptosis inhibition in donor stem cells improves hematopoietic stem cell transplantation. J. Exp. Med..

[52] Afreen S, Weiss JM, Strahm B, Erlacher M (2018). Concise review: cheating death for a better transplant. Stem cells.

[53] Silva M (1998). Expression of Bcl-x in erythroid precursors from patients with polycythemia vera. N. Engl. J. Med..

