# Supplementary figures and images for: BCL-XL expression is essential for human erythropoiesis and engraftment of hematopoietic stem cells
Source: Cell Death Dis. 2020 Jan 6;11(1):8. doi: 10.1038/s41419-019-2203-z (PMC6944703; doi:10.1038/s41419-019-2203-z)

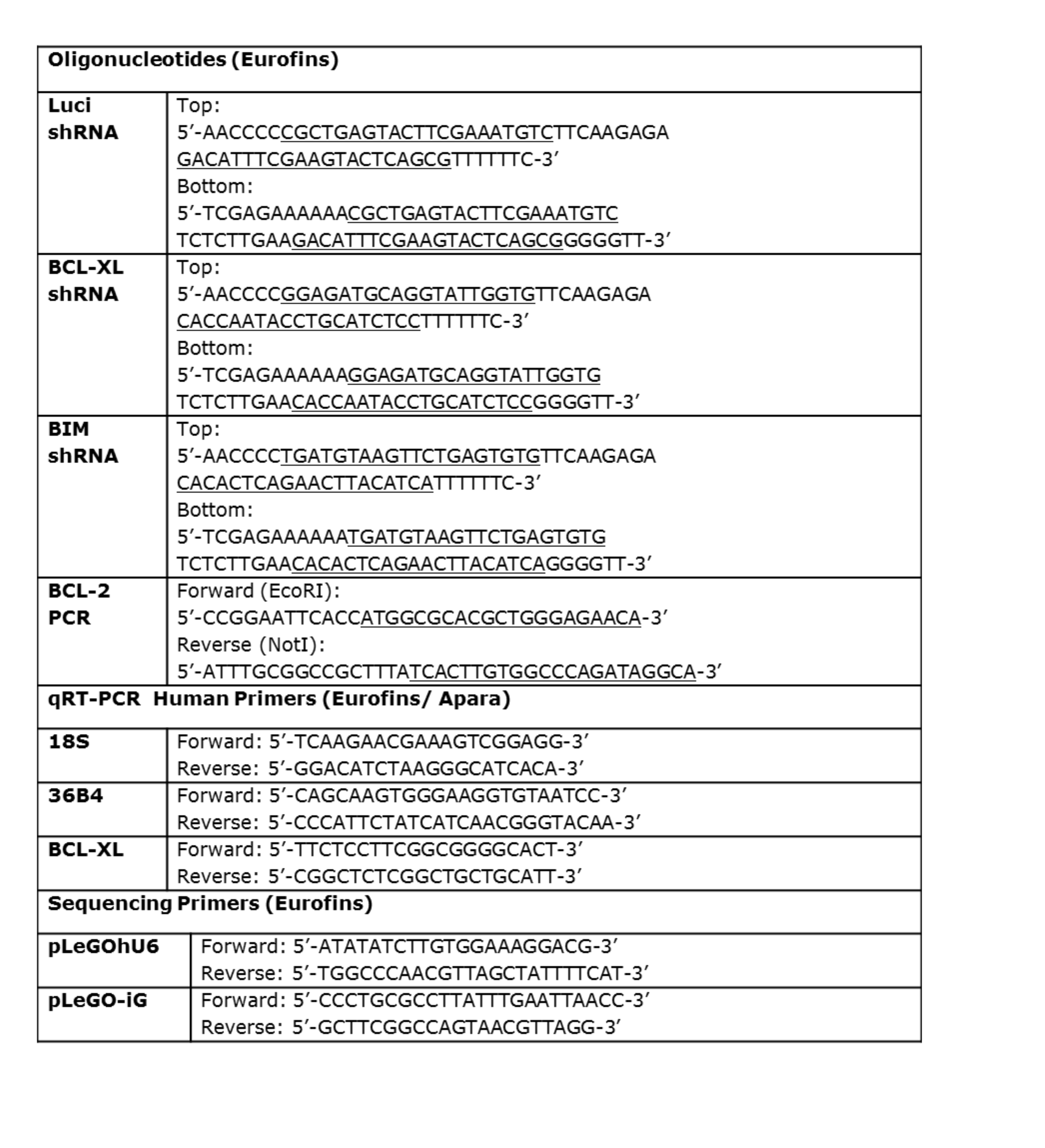

Supplement: Supplementary file 2 — Suppl. Table 1 [file 41419_2019_2203_MOESM2_ESM.png]

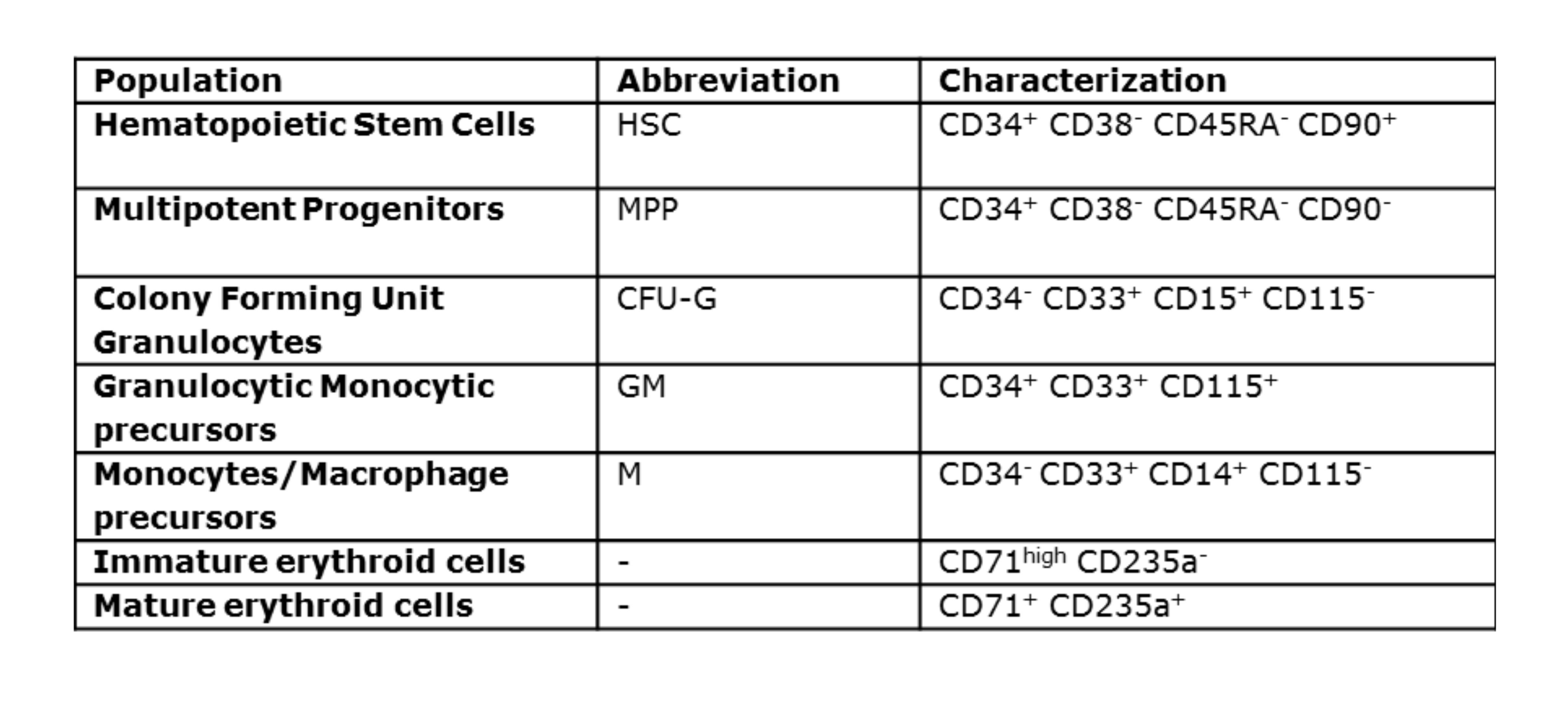

Supplement: Supplementary file 3 — Suppl. Table 2 [file 41419_2019_2203_MOESM3_ESM.png]

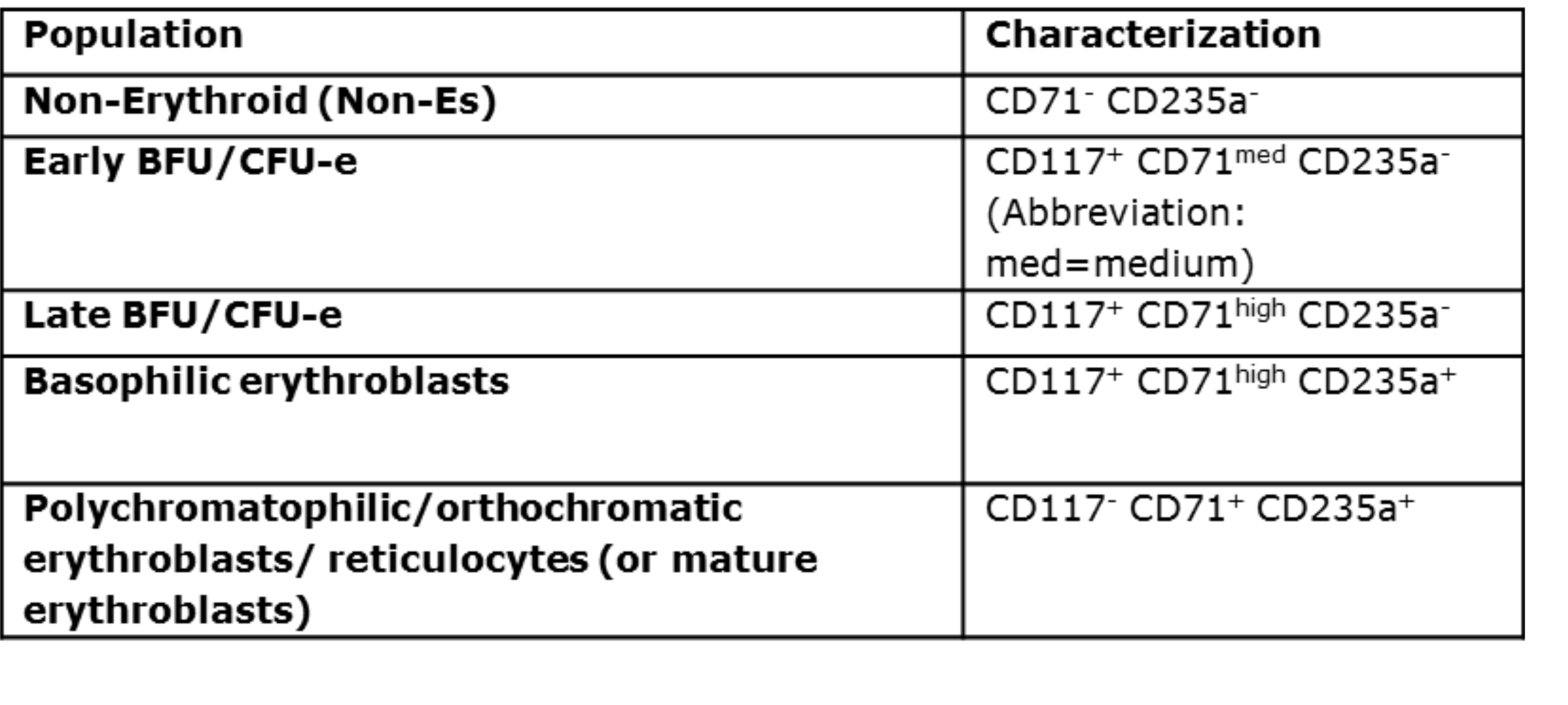

Supplement: Supplementary file 4 — Suppl. Table 3 [file 41419_2019_2203_MOESM4_ESM.png]

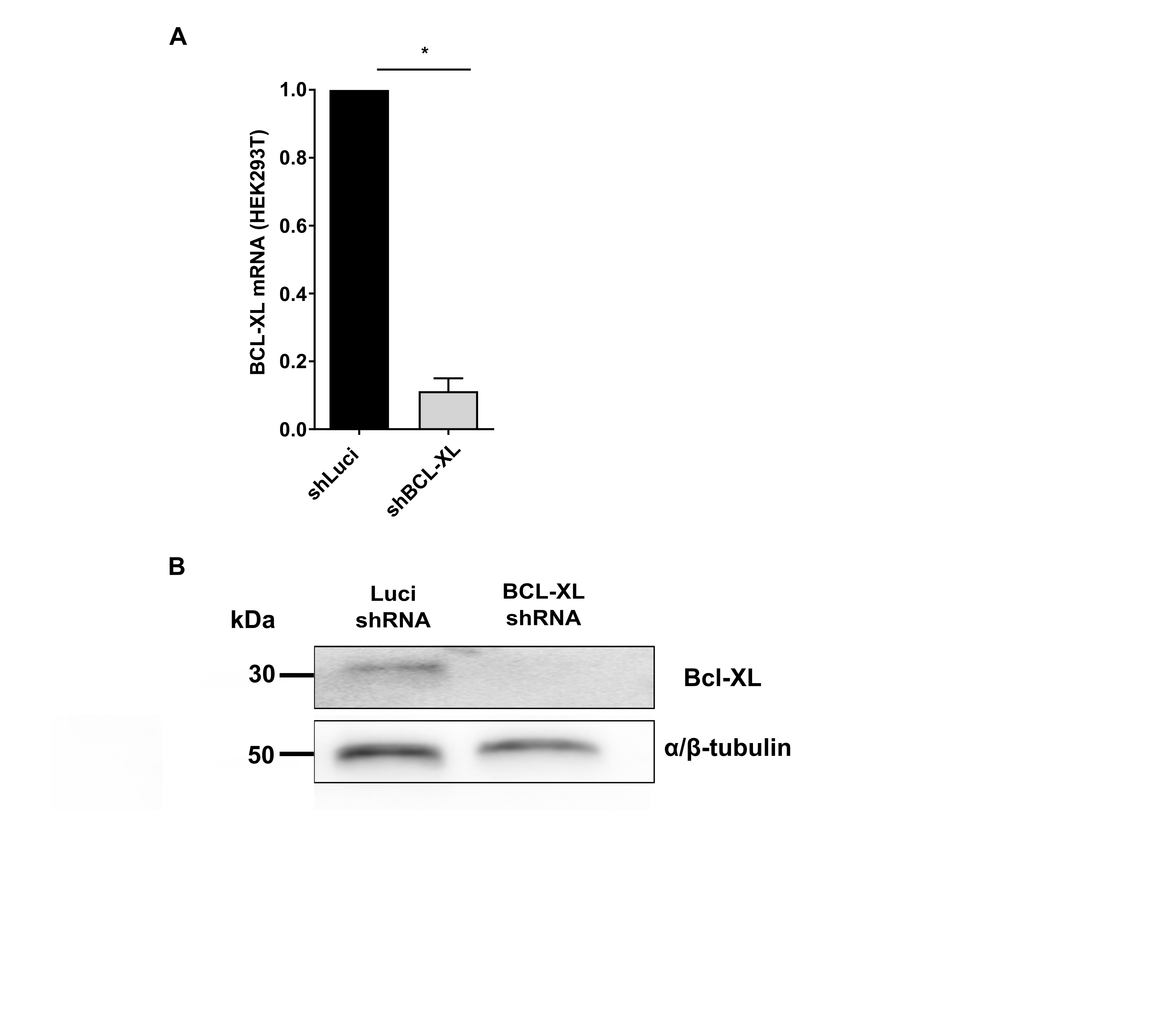

Supplement: Supplementary file 5 — Suppl. Figure 1 [file 41419_2019_2203_MOESM5_ESM.png]

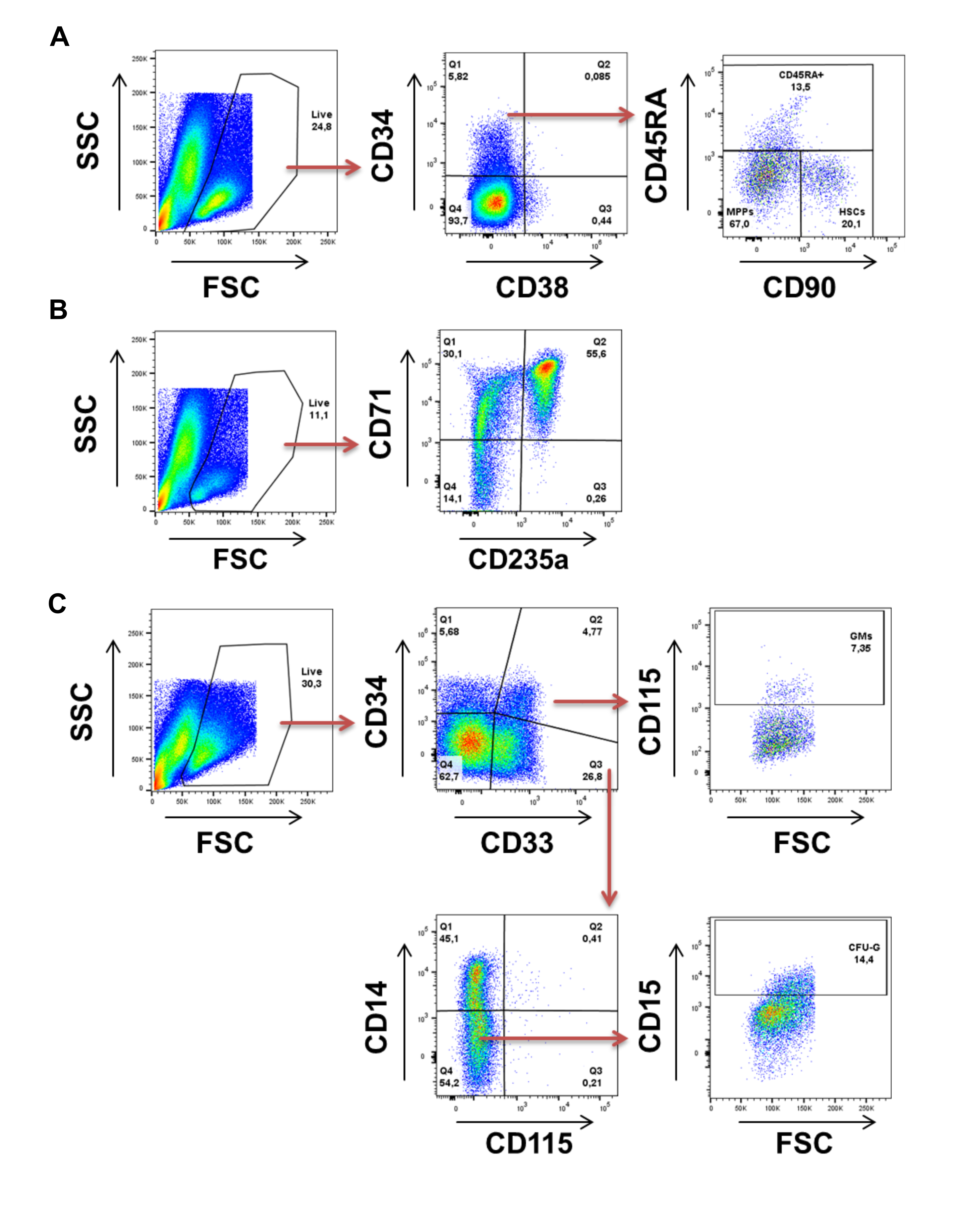

Supplement: Supplementary file 6 — Suppl. Figure 2 [file 41419_2019_2203_MOESM6_ESM.png]

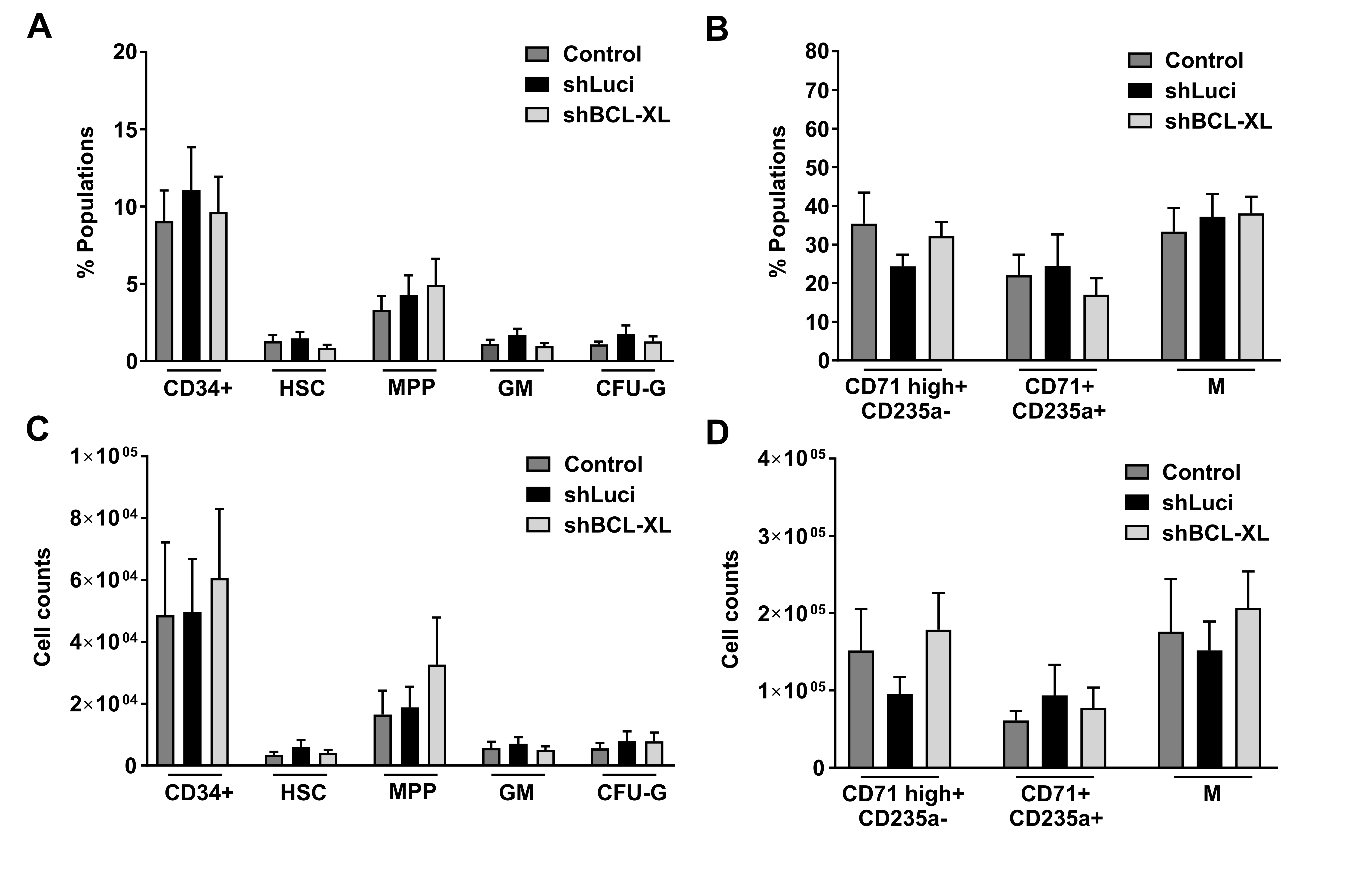

Supplement: Supplementary file 7 — Suppl. Figure 3 [file 41419_2019_2203_MOESM7_ESM.png]

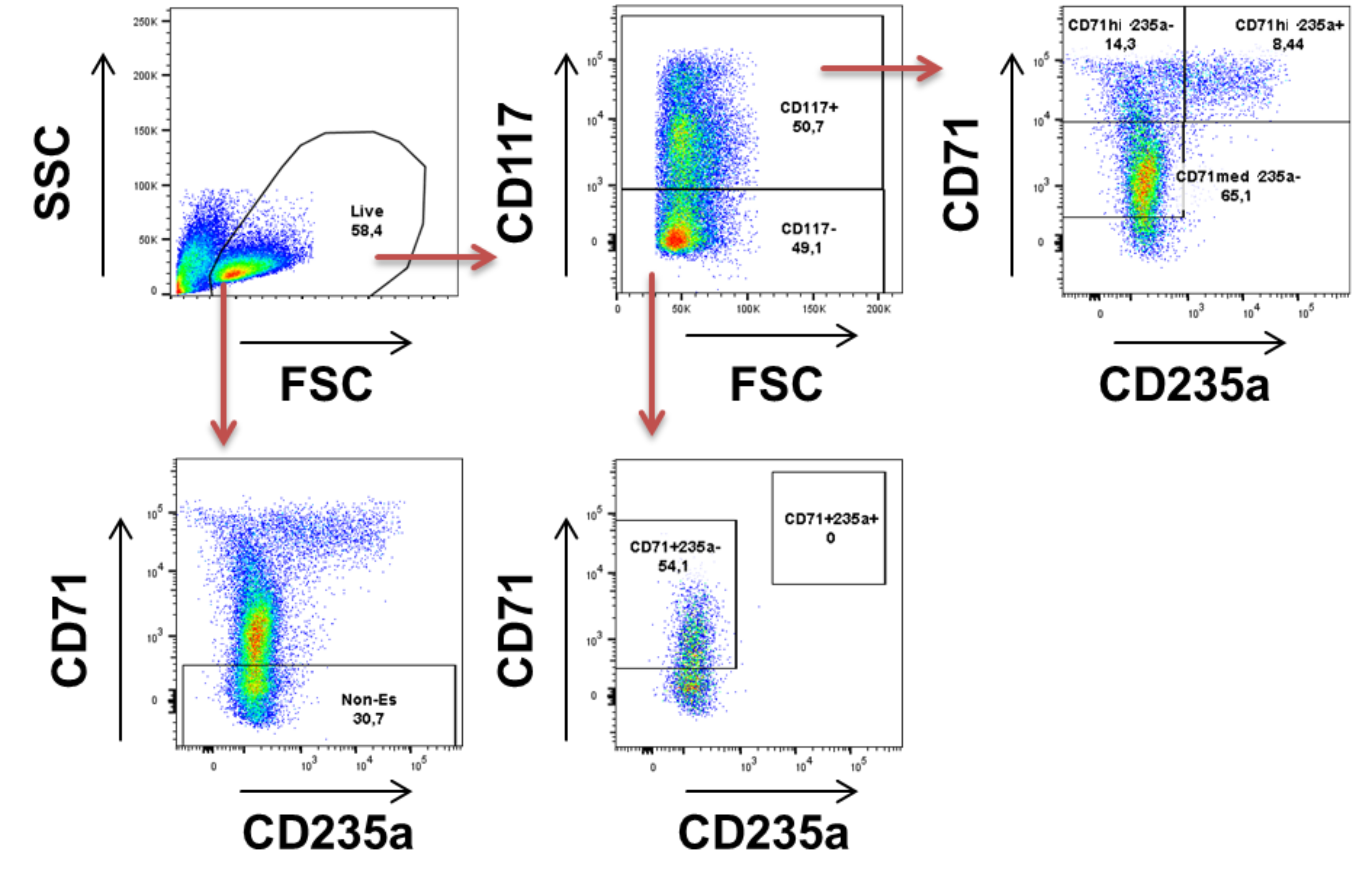

Supplement: Supplementary file 8 — Supp. Figure 4 [file 41419_2019_2203_MOESM8_ESM.png]

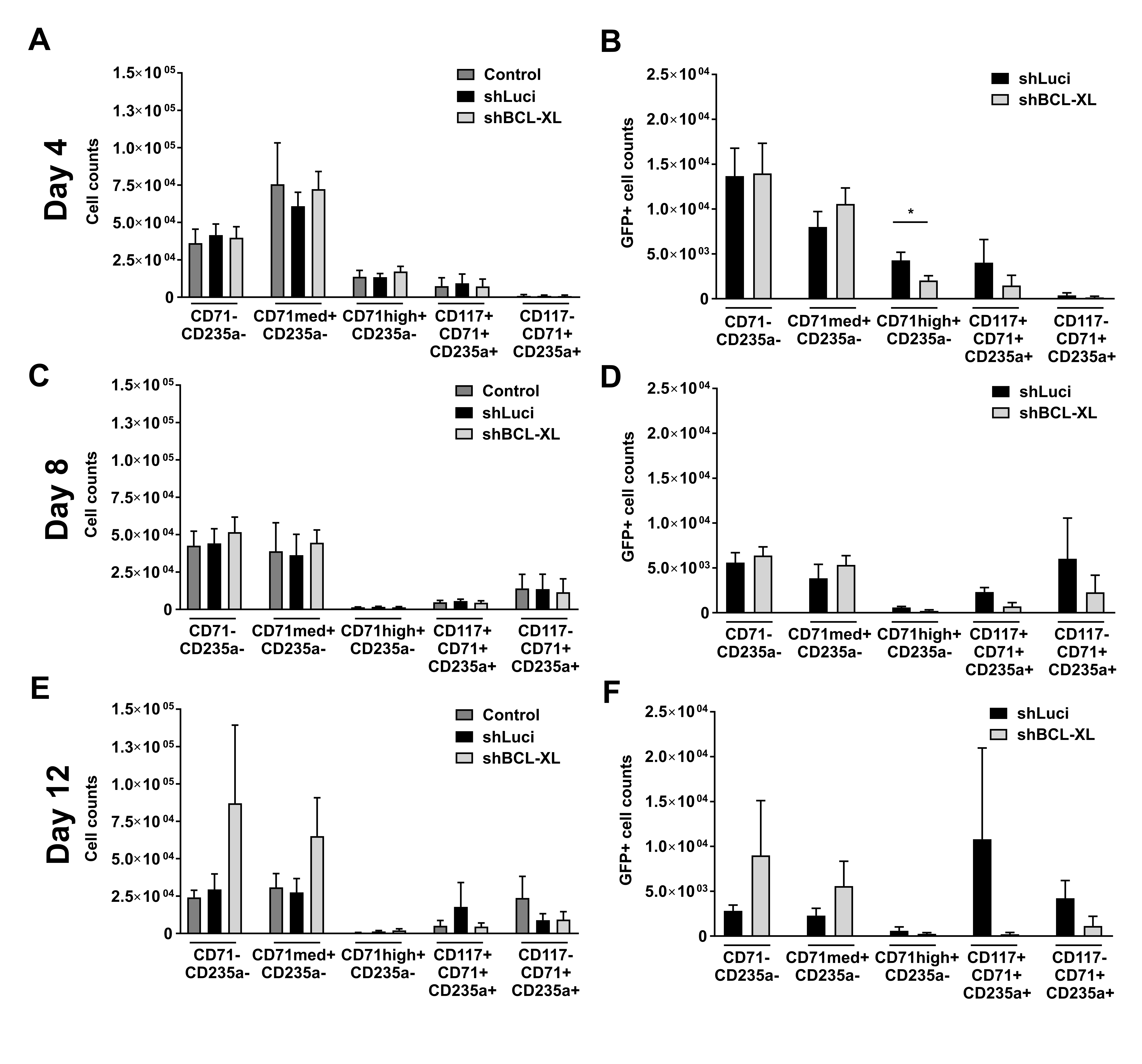

Supplement: Supplementary file 9 — Suppl. Figure 5 [file 41419_2019_2203_MOESM9_ESM.png]

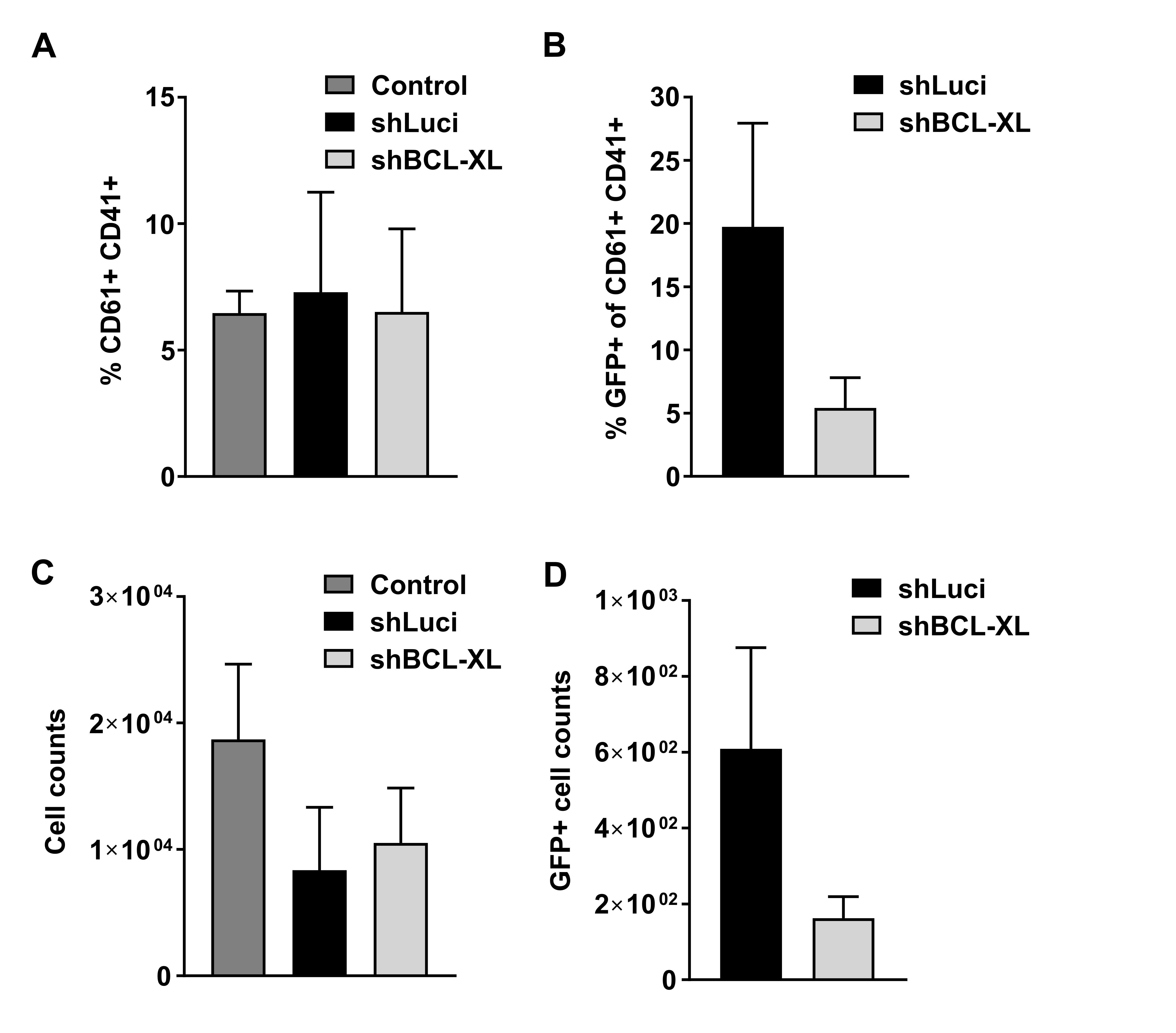

Supplement: Supplementary file 10 — Suppl. Figure 6 [file 41419_2019_2203_MOESM10_ESM.png]

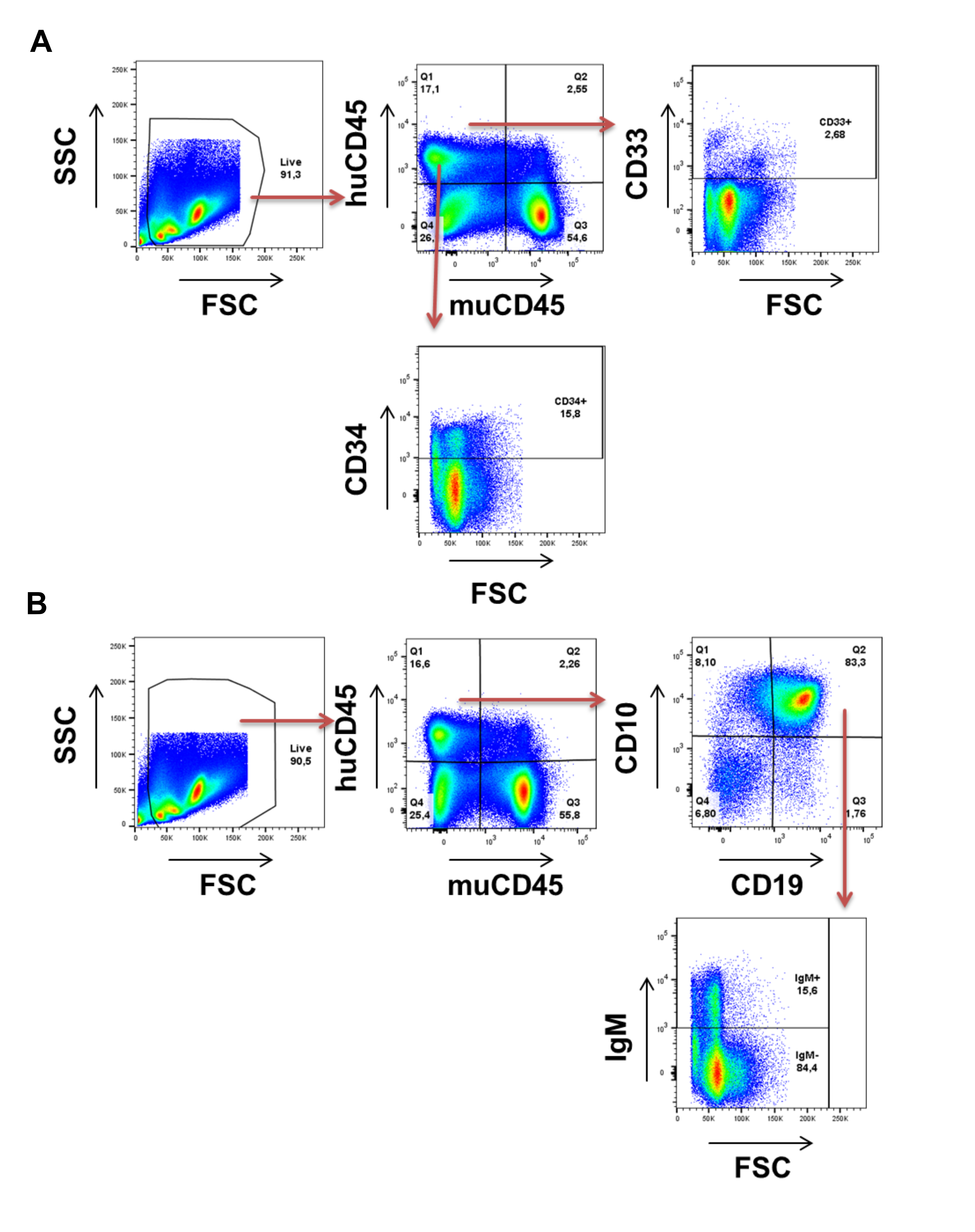

Supplement: Supplementary file 11 — Suppl. Figure 7 [file 41419_2019_2203_MOESM11_ESM.png]

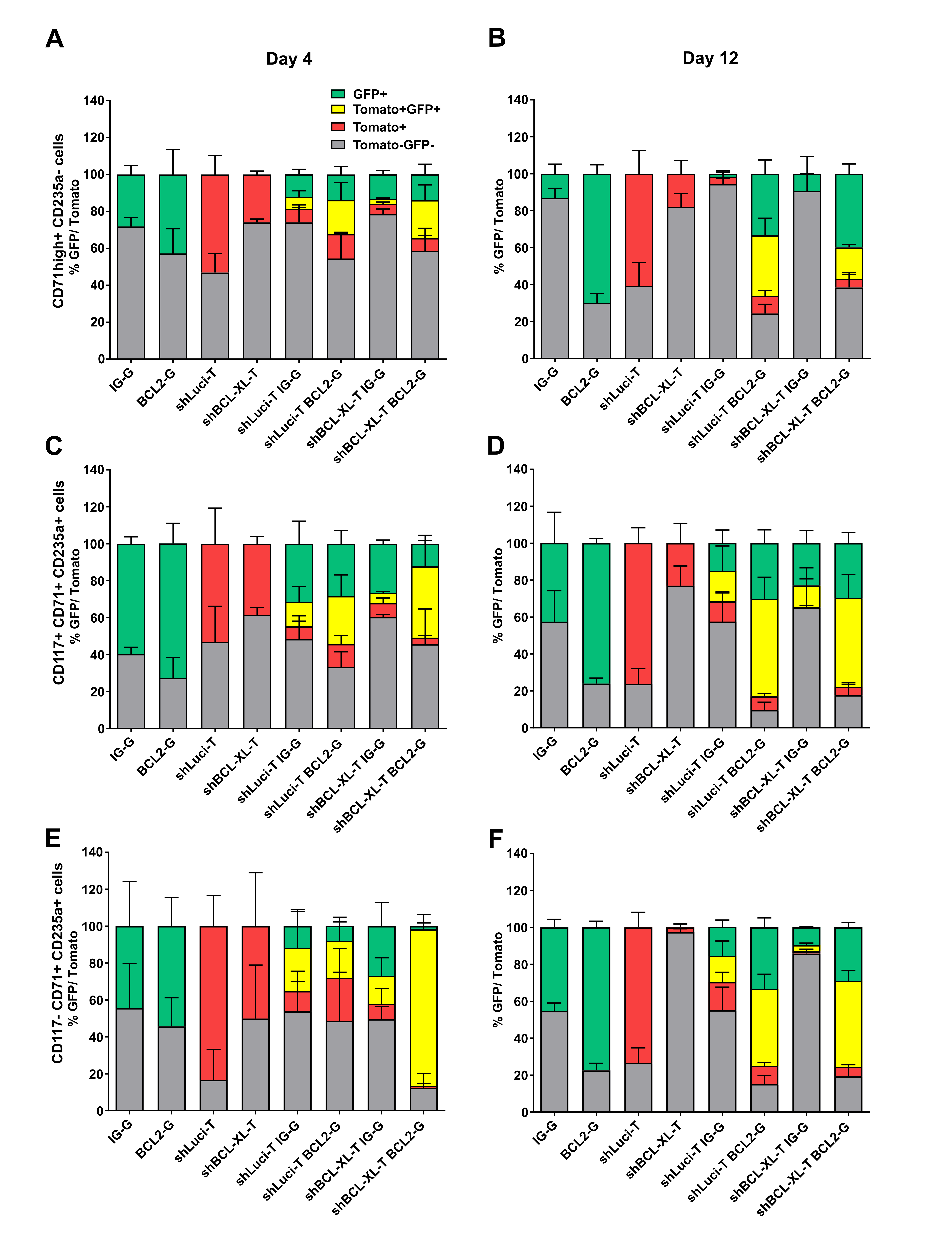

Supplement: Supplementary file 12 — Suppl. Figure 8 [file 41419_2019_2203_MOESM12_ESM.png]

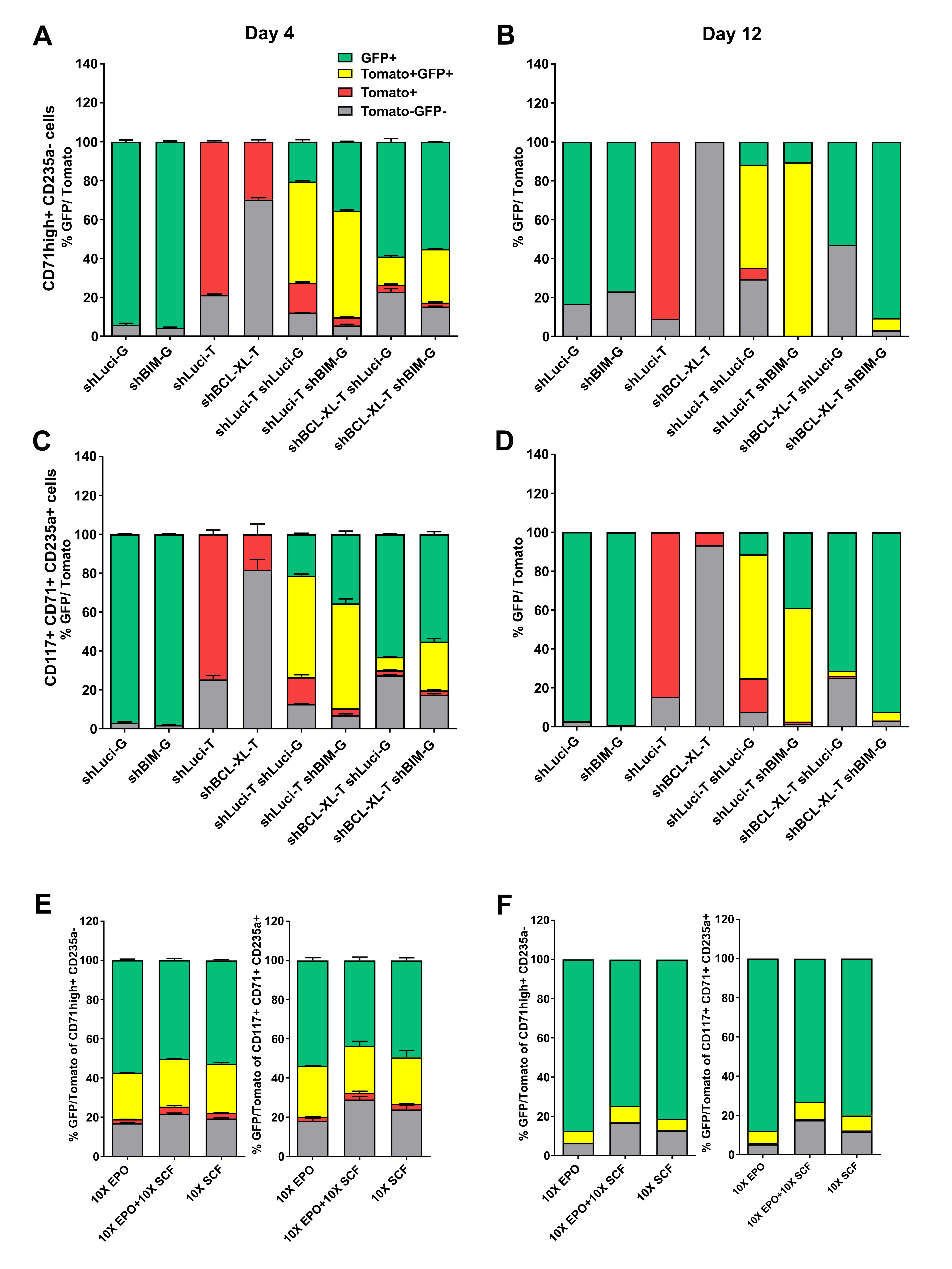

Supplement: Supplementary file 13 — Suppl. Figure 9 [file 41419_2019_2203_MOESM13_ESM.png]

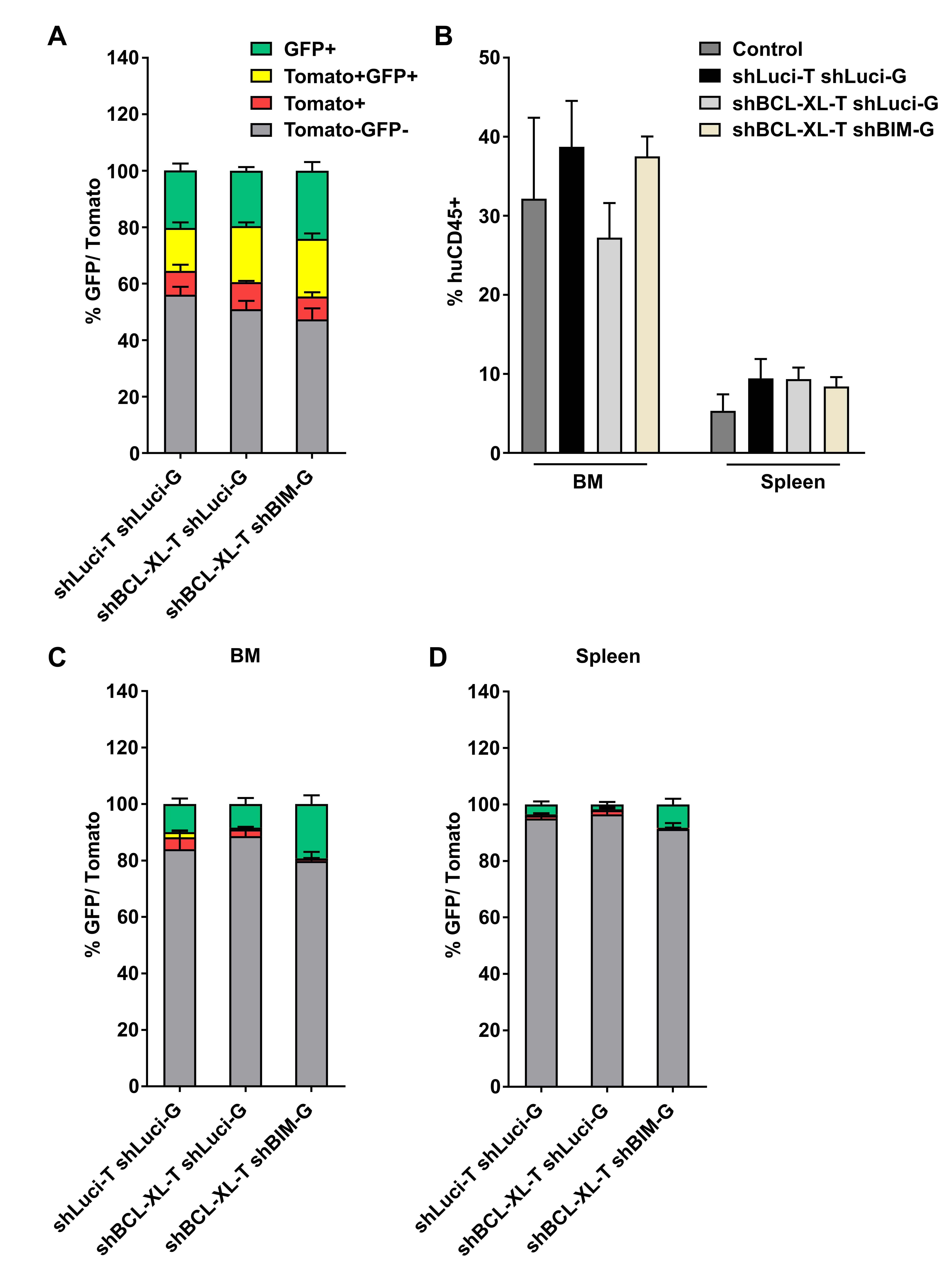

Supplement: Supplementary file 14 — Suppl. Figure 10 [file 41419_2019_2203_MOESM14_ESM.png]

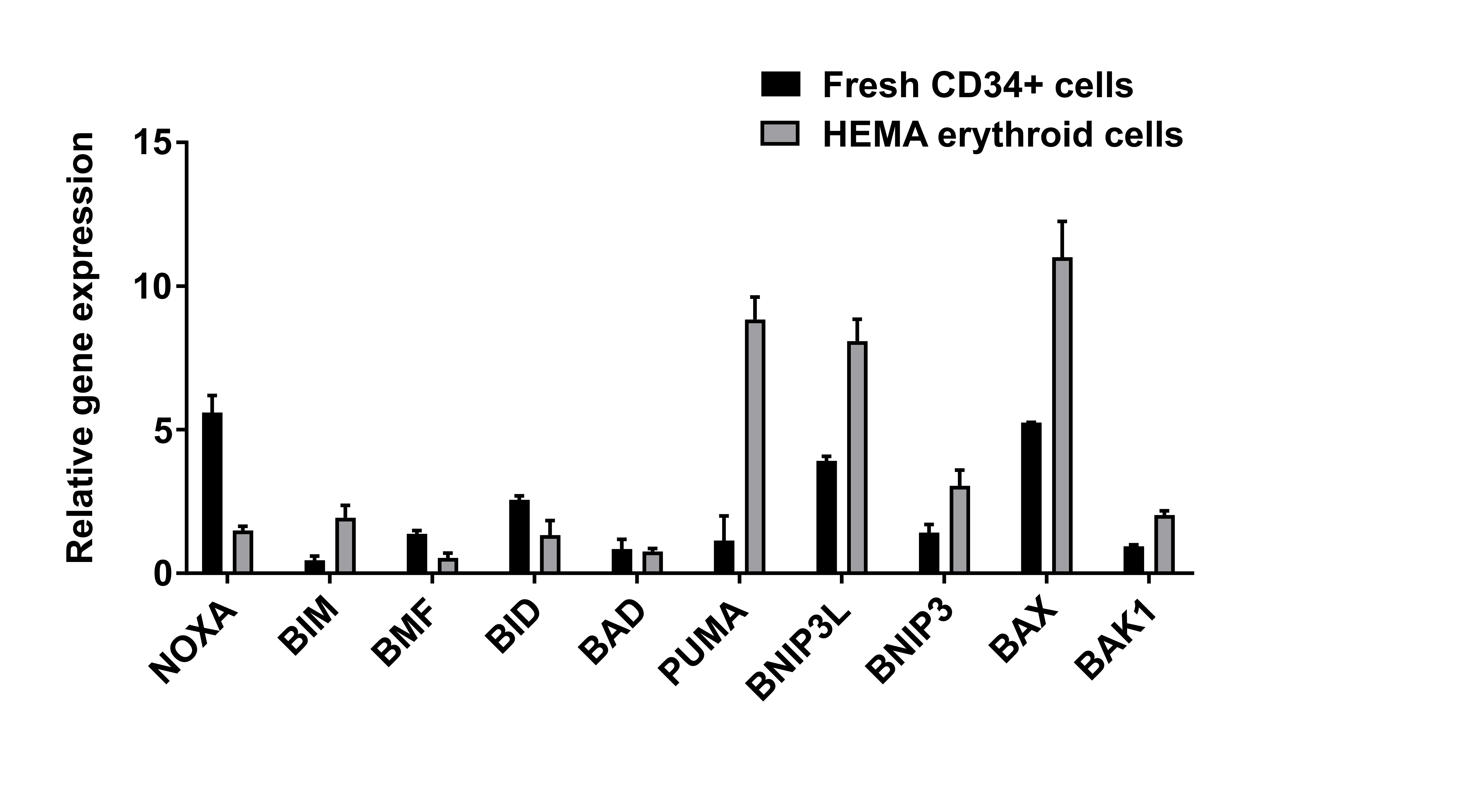

Supplement: Supplementary file 15 — Suppl. Figure 11 [file 41419_2019_2203_MOESM15_ESM.png]
