# Supplementary material for: BCL-XL expression is essential for human erythropoiesis and engraftment of hematopoietic stem cells
Source: Cell Death Dis. 2020 Jan 6;11(1):8. doi: 10.1038/s41419-019-2203-z (PMC6944703; doi:10.1038/s41419-019-2203-z)
Supplement: Supplementary file 16 — merged suppl. data [file 41419_2019_2203_MOESM16_ESM.pptx]

## Slide 1
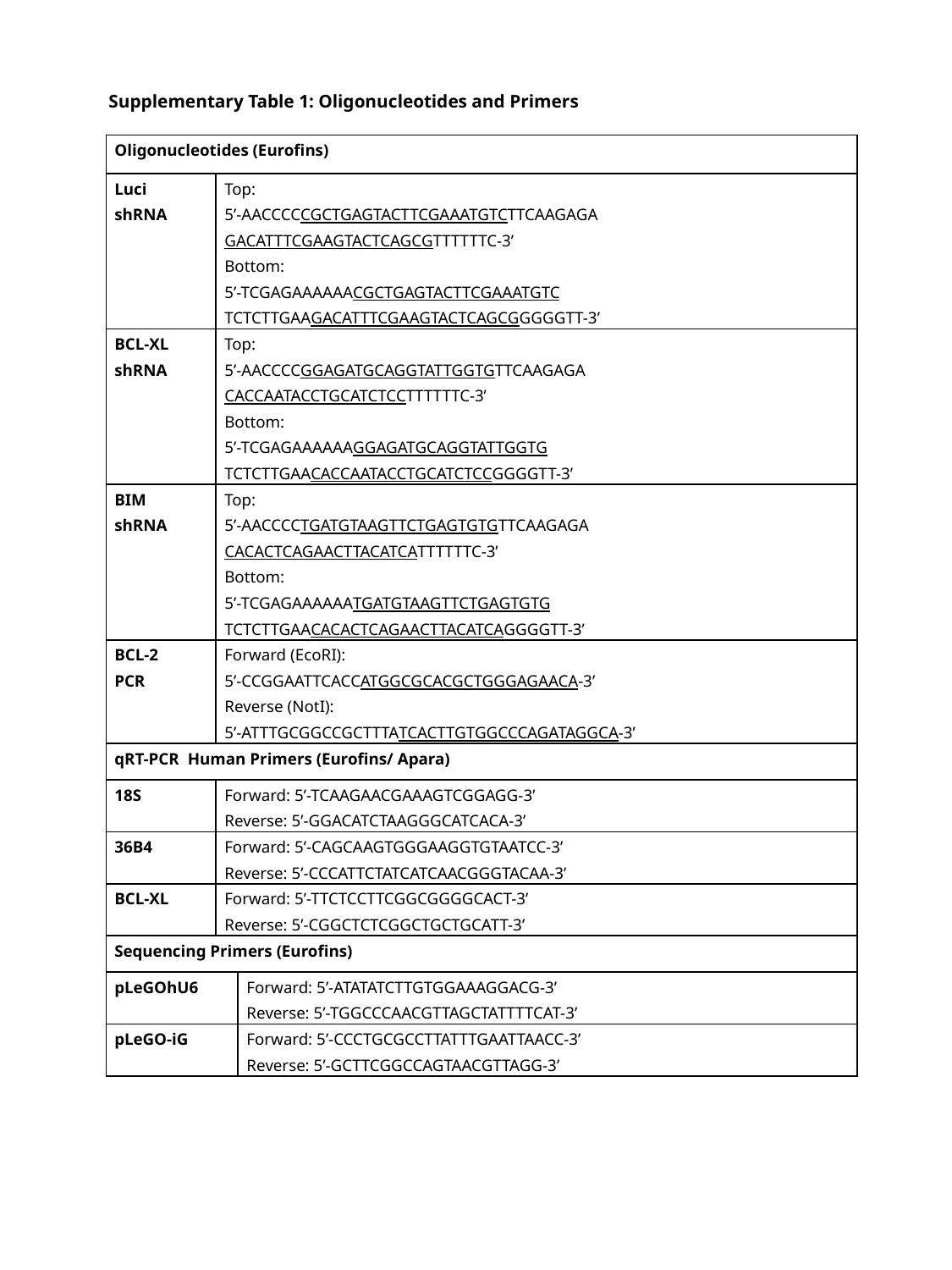

Supplementary Table 1: Oligonucleotides and Primers
| Oligonucleotides (Eurofins) | | |
| --- | --- | --- |
| Luci shRNA | Top: 5’-AACCCCCGCTGAGTACTTCGAAATGTCTTCAAGAGA GACATTTCGAAGTACTCAGCGTTTTTTC-3’ Bottom: 5’-TCGAGAAAAAACGCTGAGTACTTCGAAATGTC TCTCTTGAAGACATTTCGAAGTACTCAGCGGGGGTT-3’ | |
| BCL-XL shRNA | Top: 5’-AACCCCGGAGATGCAGGTATTGGTGTTCAAGAGA CACCAATACCTGCATCTCCTTTTTTC-3’ Bottom: 5’-TCGAGAAAAAAGGAGATGCAGGTATTGGTG TCTCTTGAACACCAATACCTGCATCTCCGGGGTT-3’ | |
| BIM shRNA | Top: 5’-AACCCCTGATGTAAGTTCTGAGTGTGTTCAAGAGA CACACTCAGAACTTACATCATTTTTTC-3’ Bottom: 5’-TCGAGAAAAAATGATGTAAGTTCTGAGTGTG TCTCTTGAACACACTCAGAACTTACATCAGGGGTT-3’ | |
| BCL-2 PCR | Forward (EcoRI): 5’-CCGGAATTCACCATGGCGCACGCTGGGAGAACA-3’ Reverse (NotI): 5’-ATTTGCGGCCGCTTTATCACTTGTGGCCCAGATAGGCA-3’ | |
| qRT-PCR Human Primers (Eurofins/ Apara) | | |
| 18S | Forward: 5’-TCAAGAACGAAAGTCGGAGG-3’ Reverse: 5’-GGACATCTAAGGGCATCACA-3’ | |
| 36B4 | Forward: 5’-CAGCAAGTGGGAAGGTGTAATCC-3’ Reverse: 5’-CCCATTCTATCATCAACGGGTACAA-3’ | |
| BCL-XL | Forward: 5’-TTCTCCTTCGGCGGGGCACT-3’ Reverse: 5’-CGGCTCTCGGCTGCTGCATT-3’ | |
| Sequencing Primers (Eurofins) | | |
| pLeGOhU6 | | Forward: 5’-ATATATCTTGTGGAAAGGACG-3’ Reverse: 5’-TGGCCCAACGTTAGCTATTTTCAT-3’ |
| pLeGO-iG | | Forward: 5’-CCCTGCGCCTTATTTGAATTAACC-3’ Reverse: 5’-GCTTCGGCCAGTAACGTTAGG-3’ |

## Slide 2
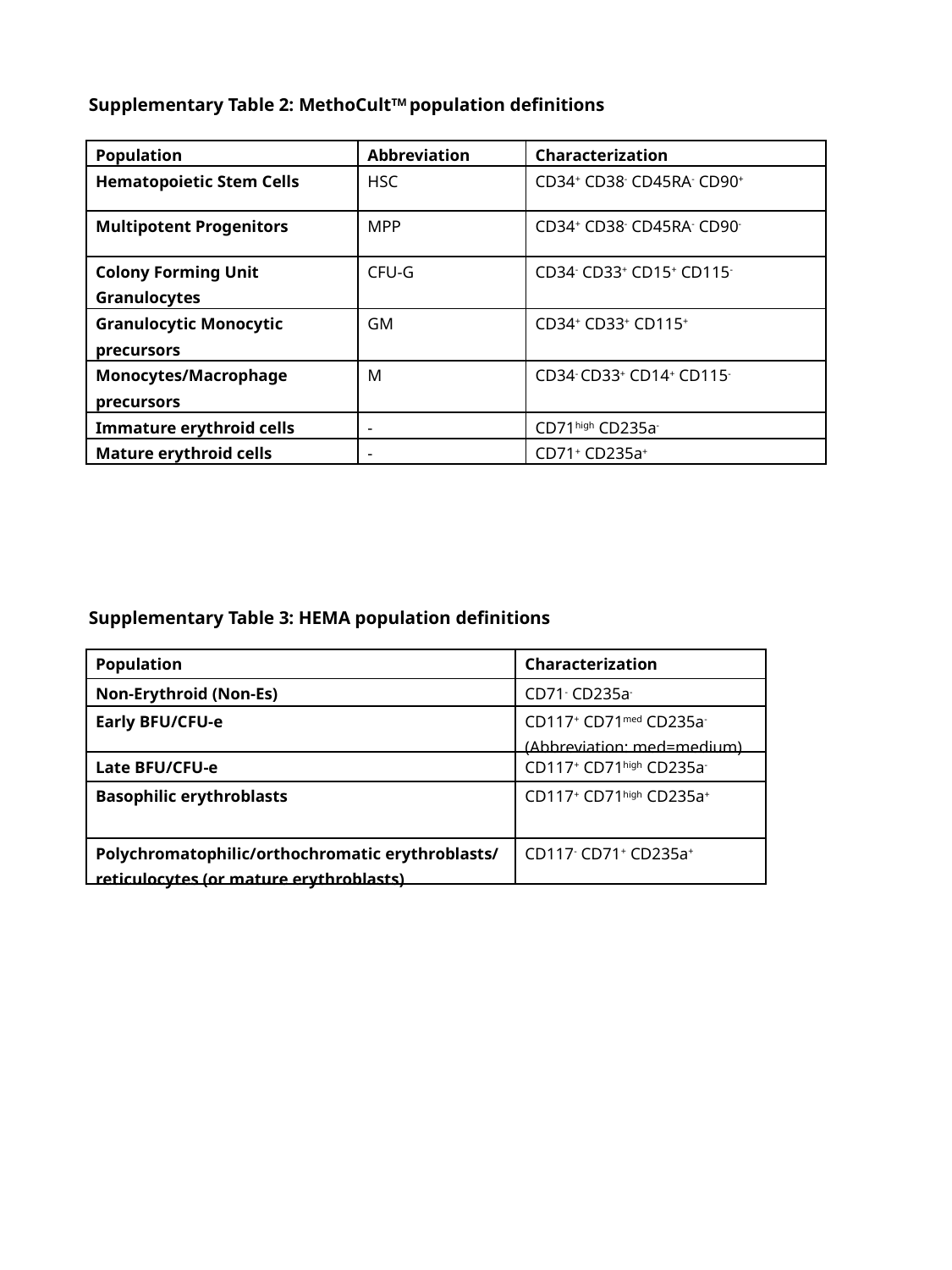

Supplementary Table 2: MethoCultTM population definitions
| Population | Abbreviation | Characterization |
| --- | --- | --- |
| Hematopoietic Stem Cells | HSC | CD34+ CD38- CD45RA- CD90+ |
| Multipotent Progenitors | MPP | CD34+ CD38- CD45RA- CD90- |
| Colony Forming Unit Granulocytes | CFU-G | CD34- CD33+ CD15+ CD115- |
| Granulocytic Monocytic precursors | GM | CD34+ CD33+ CD115+ |
| Monocytes/Macrophage precursors | M | CD34- CD33+ CD14+ CD115- |
| Immature erythroid cells | - | CD71high CD235a- |
| Mature erythroid cells | - | CD71+ CD235a+ |
Supplementary Table 3: HEMA population definitions
| Population | Characterization |
| --- | --- |
| Non-Erythroid (Non-Es) | CD71- CD235a- |
| Early BFU/CFU-e | CD117+ CD71med CD235a- (Abbreviation: med=medium) |
| Late BFU/CFU-e | CD117+ CD71high CD235a- |
| Basophilic erythroblasts | CD117+ CD71high CD235a+ |
| Polychromatophilic/orthochromatic erythroblasts/ reticulocytes (or mature erythroblasts) | CD117- CD71+ CD235a+ |

## Slide 3
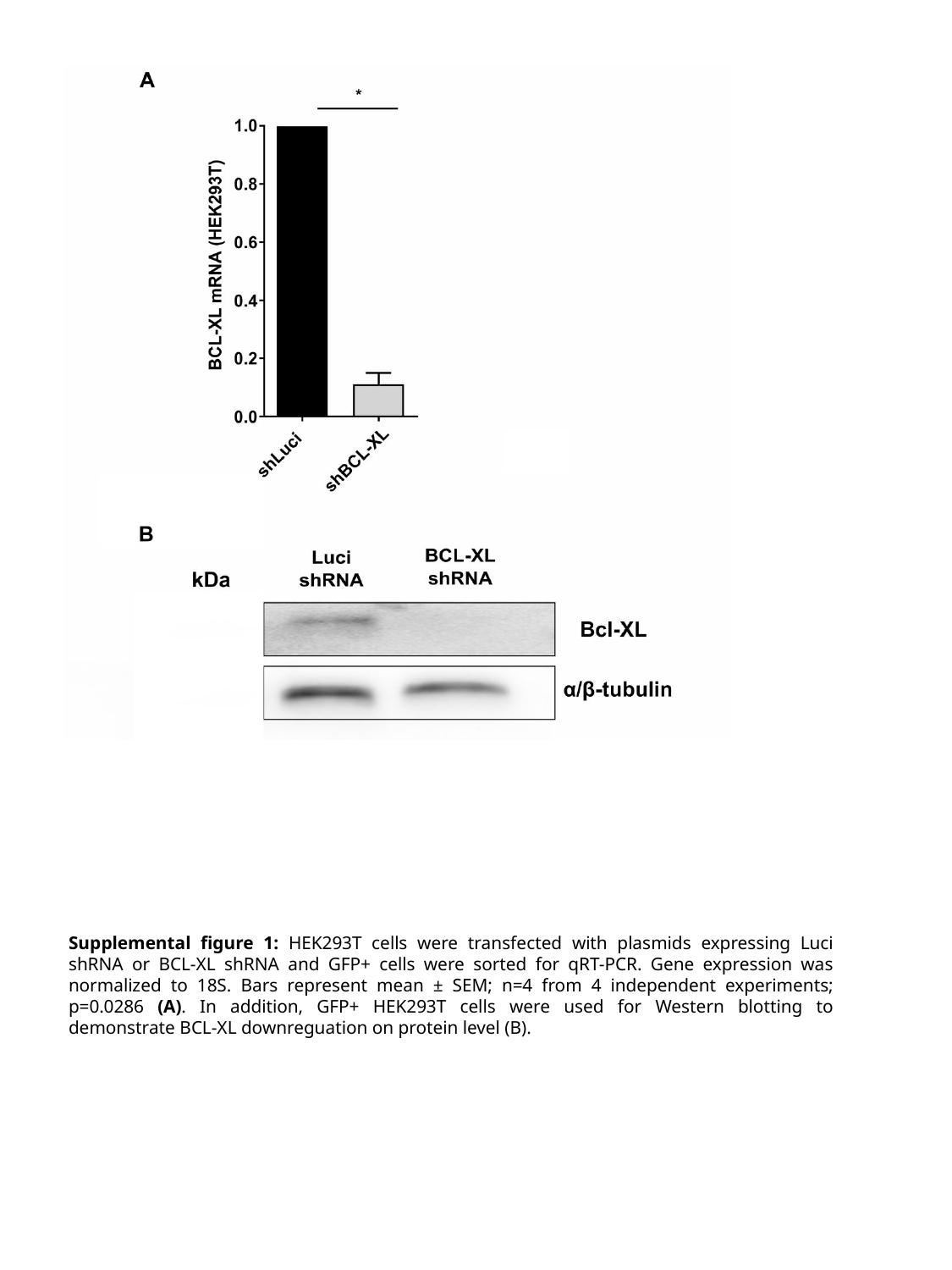

Supplemental figure 1: HEK293T cells were transfected with plasmids expressing Luci shRNA or BCL-XL shRNA and GFP+ cells were sorted for qRT-PCR. Gene expression was normalized to 18S. Bars represent mean ± SEM; n=4 from 4 independent experiments; p=0.0286 (A). In addition, GFP+ HEK293T cells were used for Western blotting to demonstrate BCL-XL downreguation on protein level (B).

## Slide 4
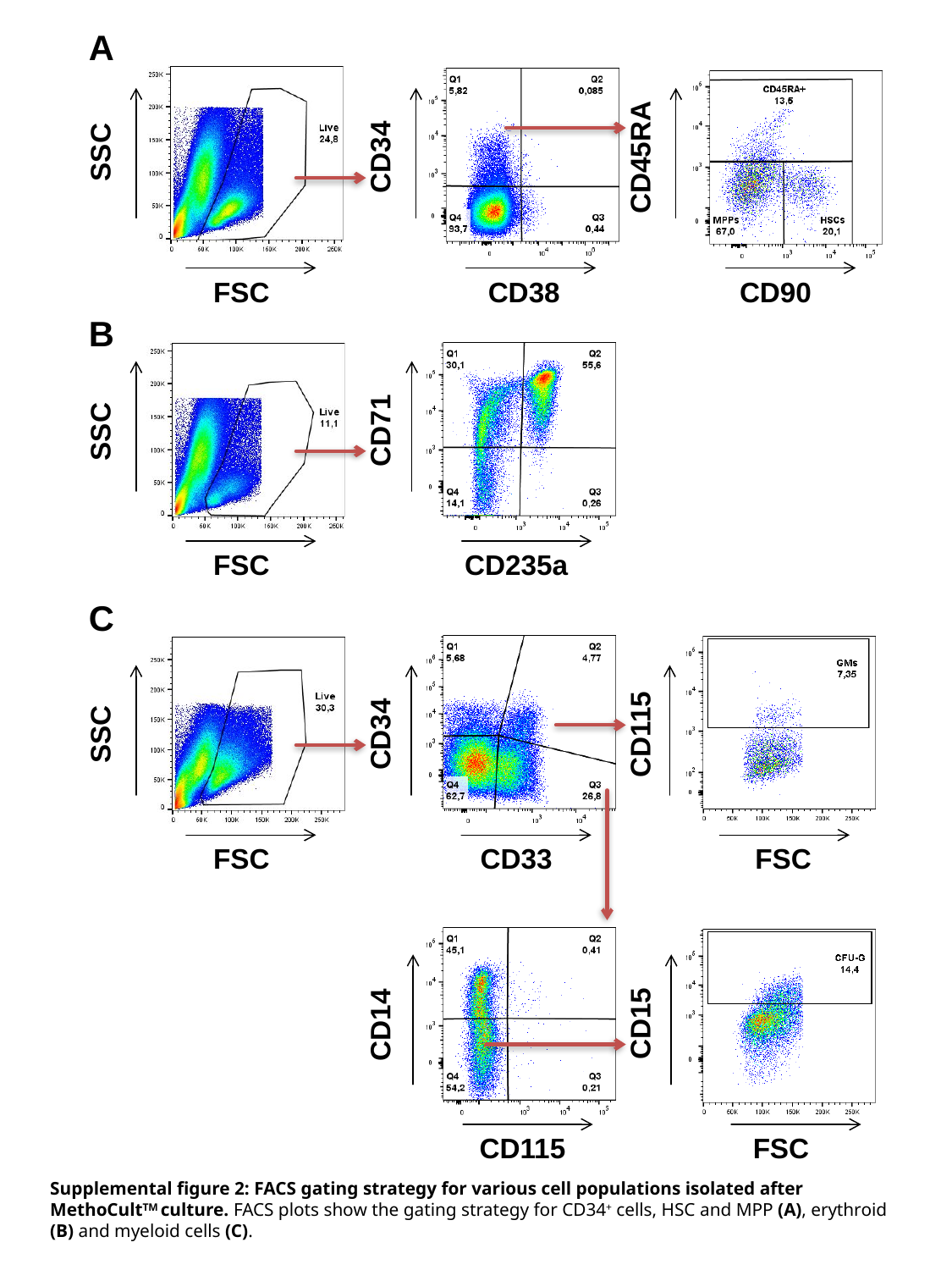

A
CD45RA
SSC
CD34
 FSC CD38 CD90
B
CD71
SSC
 FSC CD235a
C
SSC
CD34
CD115
 FSC CD33 FSC
CD15
CD14
 CD115 FSC
Supplemental figure 2: FACS gating strategy for various cell populations isolated after MethoCultTM culture. FACS plots show the gating strategy for CD34+ cells, HSC and MPP (A), erythroid (B) and myeloid cells (C).

## Slide 5
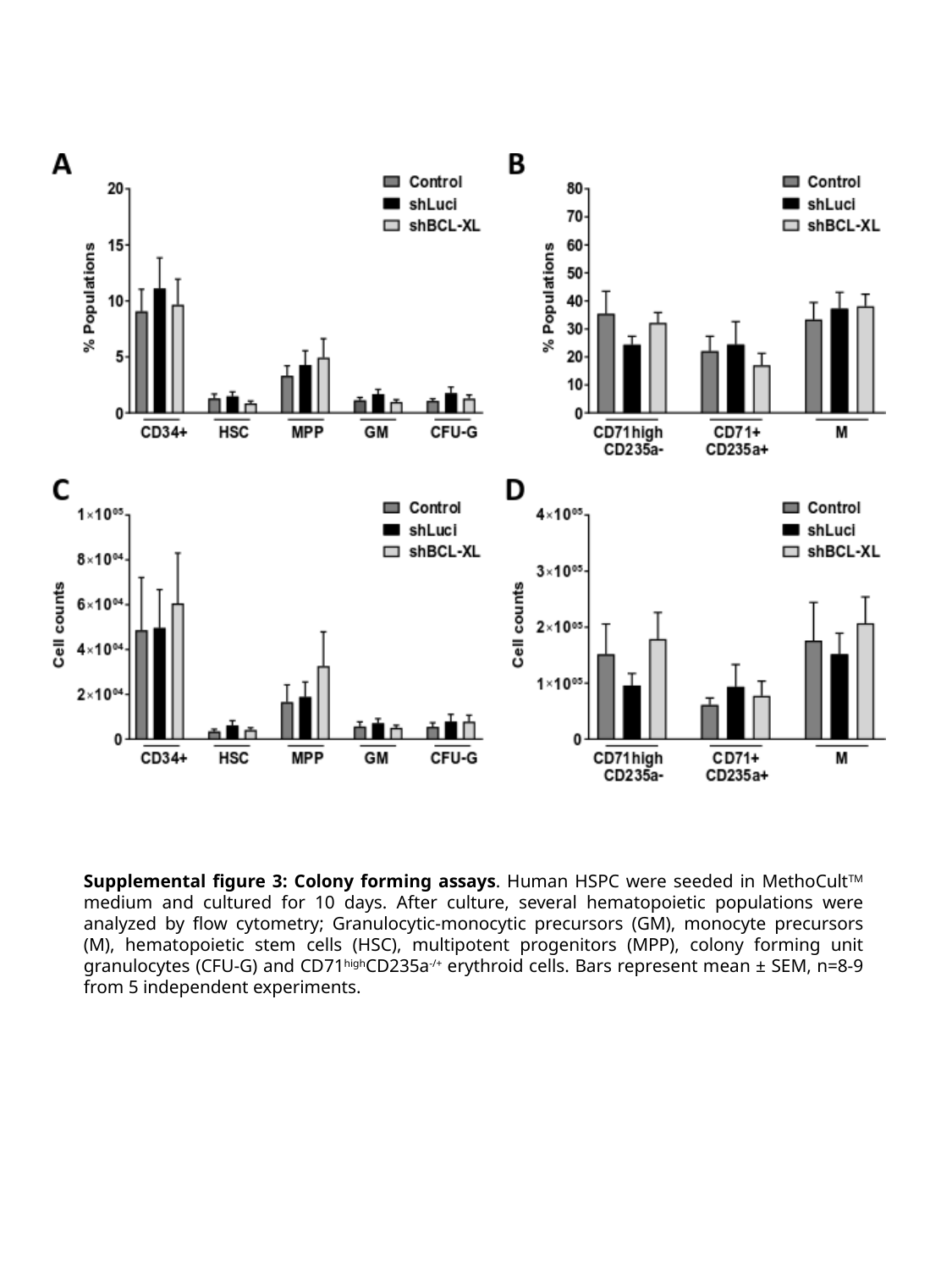

Supplemental figure 3: Colony forming assays. Human HSPC were seeded in MethoCultTM medium and cultured for 10 days. After culture, several hematopoietic populations were analyzed by flow cytometry; Granulocytic-monocytic precursors (GM), monocyte precursors (M), hematopoietic stem cells (HSC), multipotent progenitors (MPP), colony forming unit granulocytes (CFU-G) and CD71highCD235a-/+ erythroid cells. Bars represent mean ± SEM, n=8-9 from 5 independent experiments.

## Slide 6
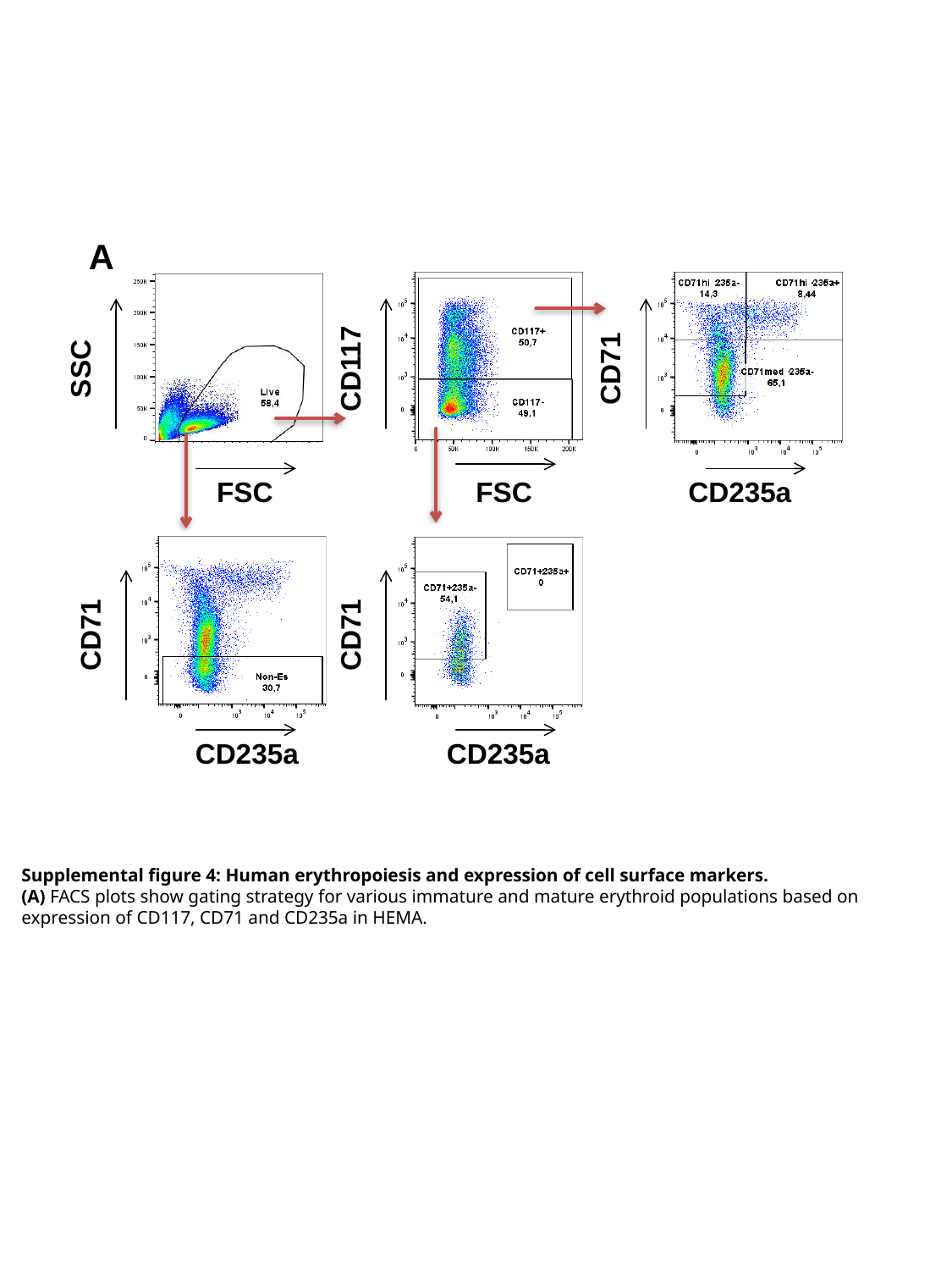

A
SSC
CD117
CD71
 FSC FSC CD235a
CD71
CD71
 CD235a CD235a
Supplemental figure 4: Human erythropoiesis and expression of cell surface markers.
(A) FACS plots show gating strategy for various immature and mature erythroid populations based on expression of CD117, CD71 and CD235a in HEMA.

## Slide 7
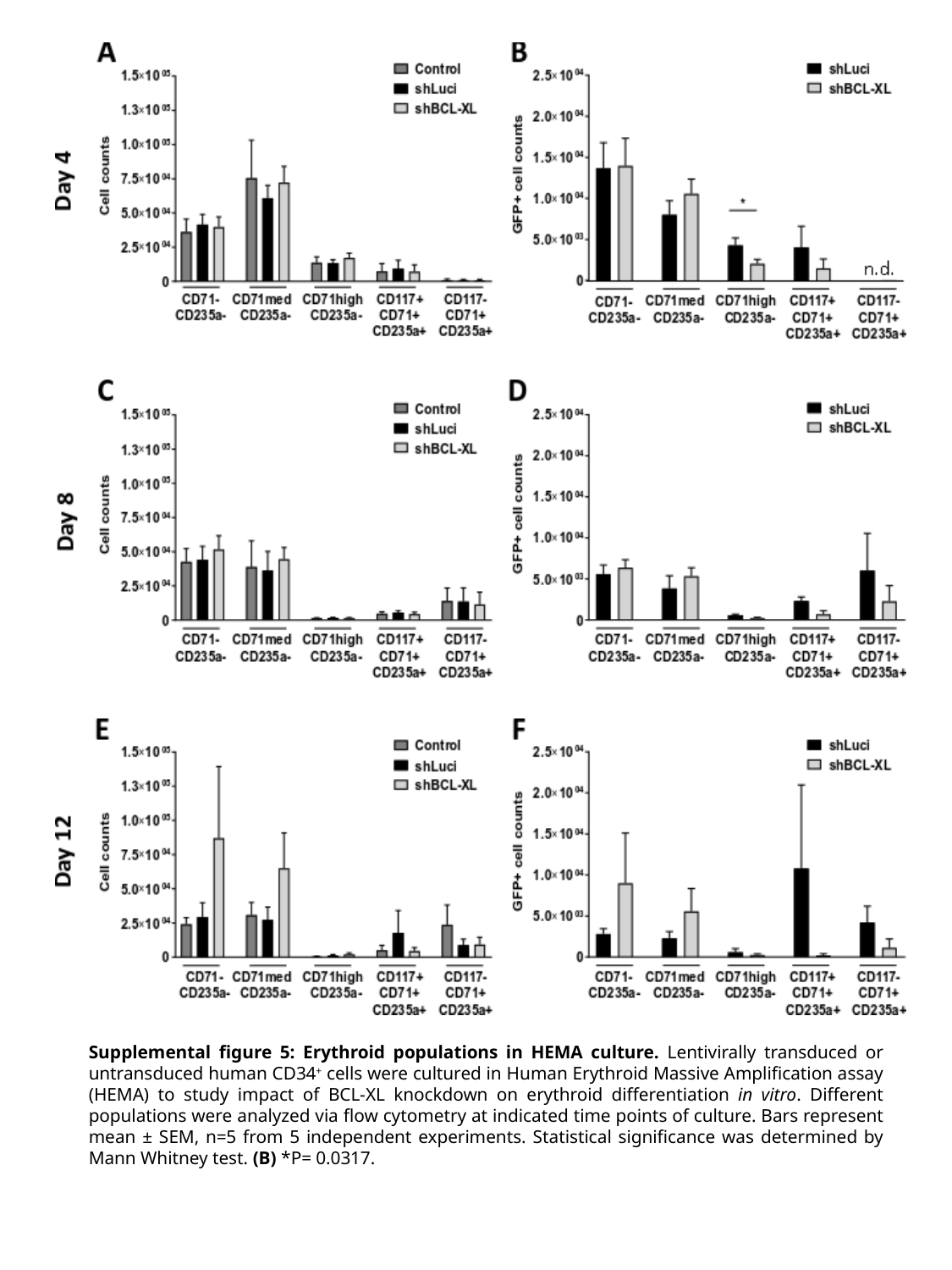

Supplemental figure 5: Erythroid populations in HEMA culture. Lentivirally transduced or untransduced human CD34+ cells were cultured in Human Erythroid Massive Amplification assay (HEMA) to study impact of BCL-XL knockdown on erythroid differentiation in vitro. Different populations were analyzed via flow cytometry at indicated time points of culture. Bars represent mean ± SEM, n=5 from 5 independent experiments. Statistical significance was determined by Mann Whitney test. (B) *P= 0.0317.

## Slide 8
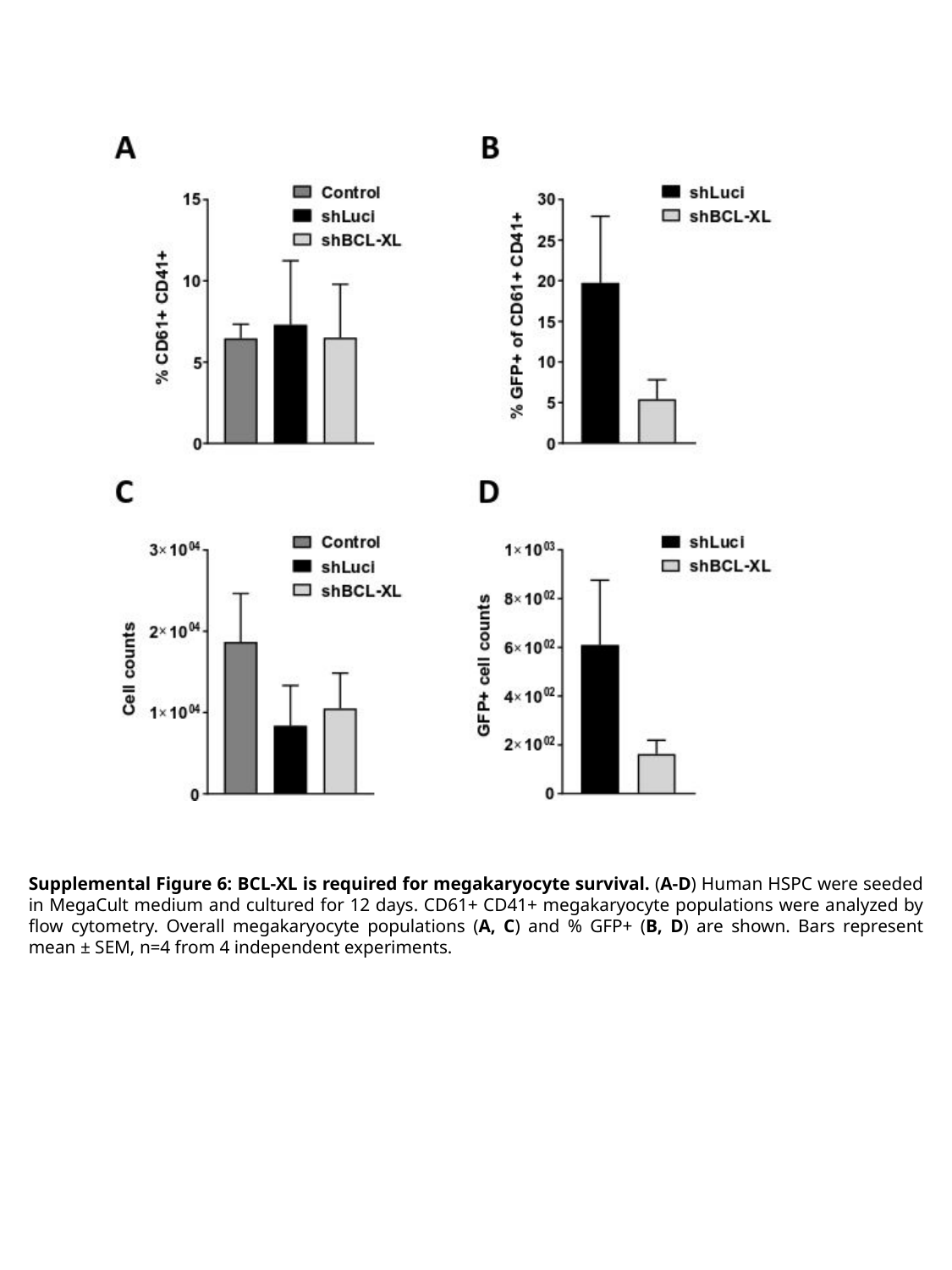

Supplemental Figure 6: BCL-XL is required for megakaryocyte survival. (A-D) Human HSPC were seeded in MegaCult medium and cultured for 12 days. CD61+ CD41+ megakaryocyte populations were analyzed by flow cytometry. Overall megakaryocyte populations (A, C) and % GFP+ (B, D) are shown. Bars represent mean ± SEM, n=4 from 4 independent experiments.

## Slide 9
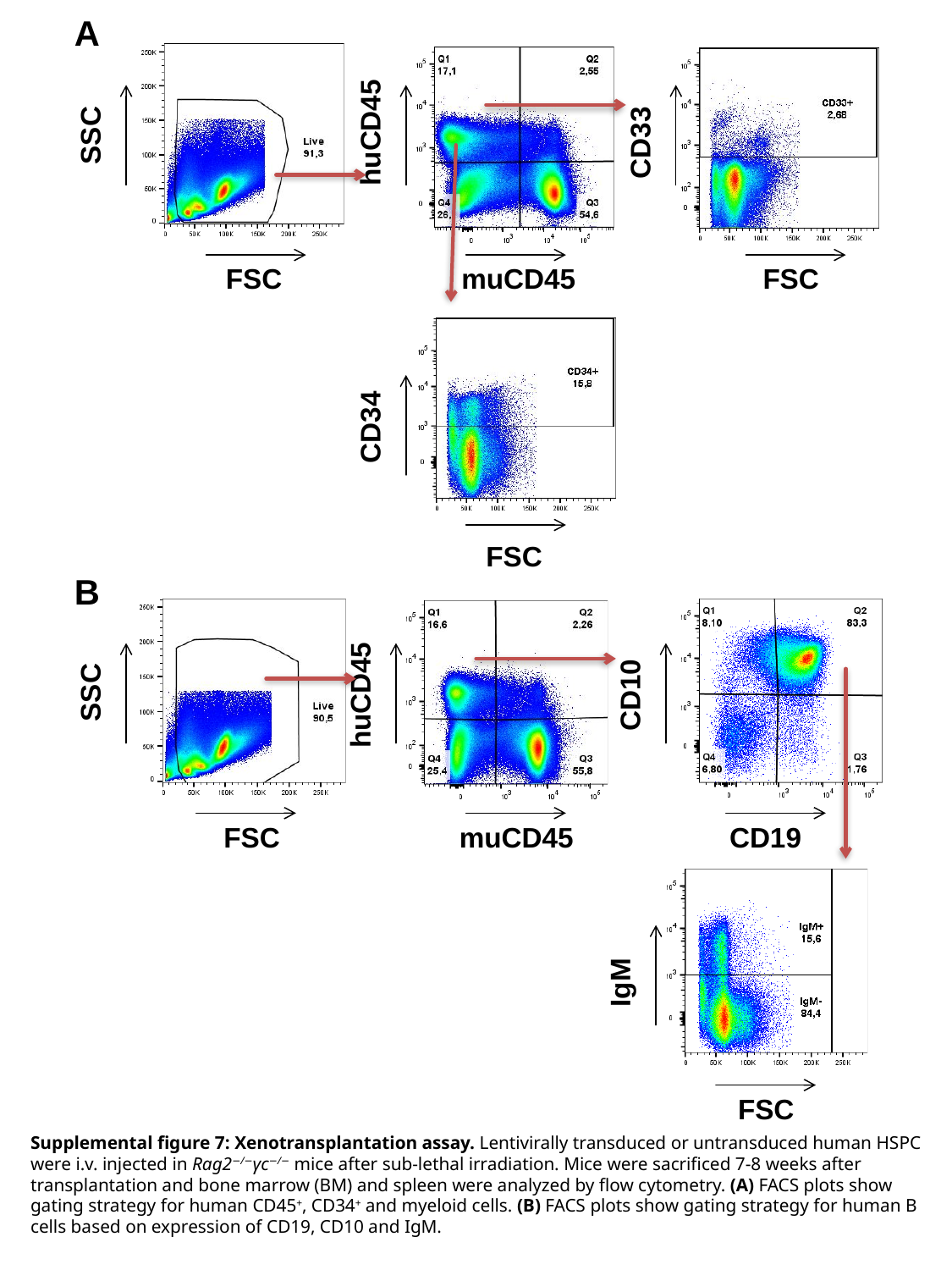

A
huCD45
CD33
SSC
 FSC muCD45 FSC
CD34
 FSC
B
huCD45
CD10
SSC
 FSC muCD45 CD19
IgM
 FSC
Supplemental figure 7: Xenotransplantation assay. Lentivirally transduced or untransduced human HSPC were i.v. injected in Rag2−/−γc−/− mice after sub-lethal irradiation. Mice were sacrificed 7-8 weeks after transplantation and bone marrow (BM) and spleen were analyzed by flow cytometry. (A) FACS plots show gating strategy for human CD45+, CD34+ and myeloid cells. (B) FACS plots show gating strategy for human B cells based on expression of CD19, CD10 and IgM.

## Slide 10
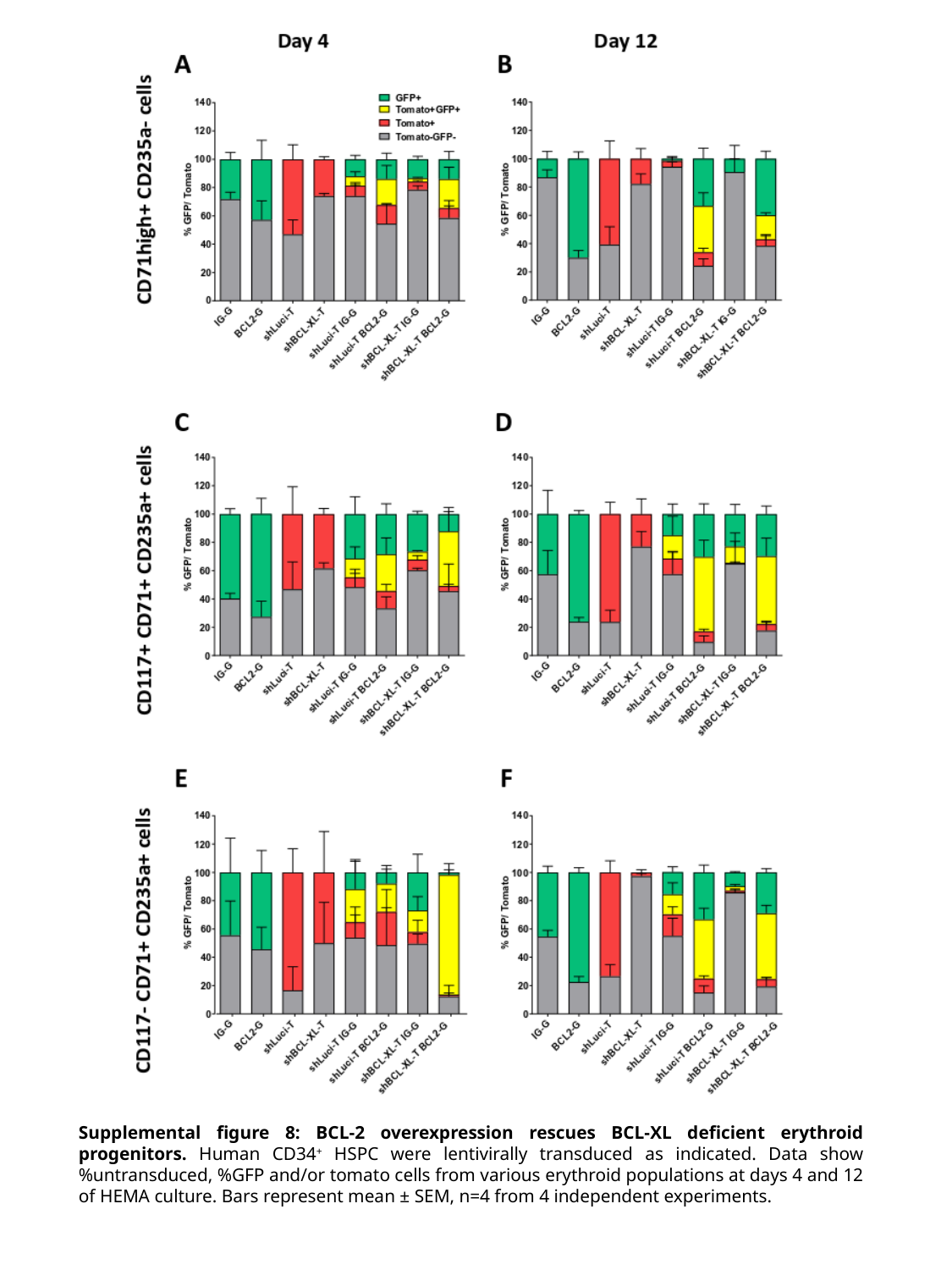

Supplemental figure 8: BCL-2 overexpression rescues BCL-XL deficient erythroid progenitors. Human CD34+ HSPC were lentivirally transduced as indicated. Data show %untransduced, %GFP and/or tomato cells from various erythroid populations at days 4 and 12 of HEMA culture. Bars represent mean ± SEM, n=4 from 4 independent experiments.

## Slide 11
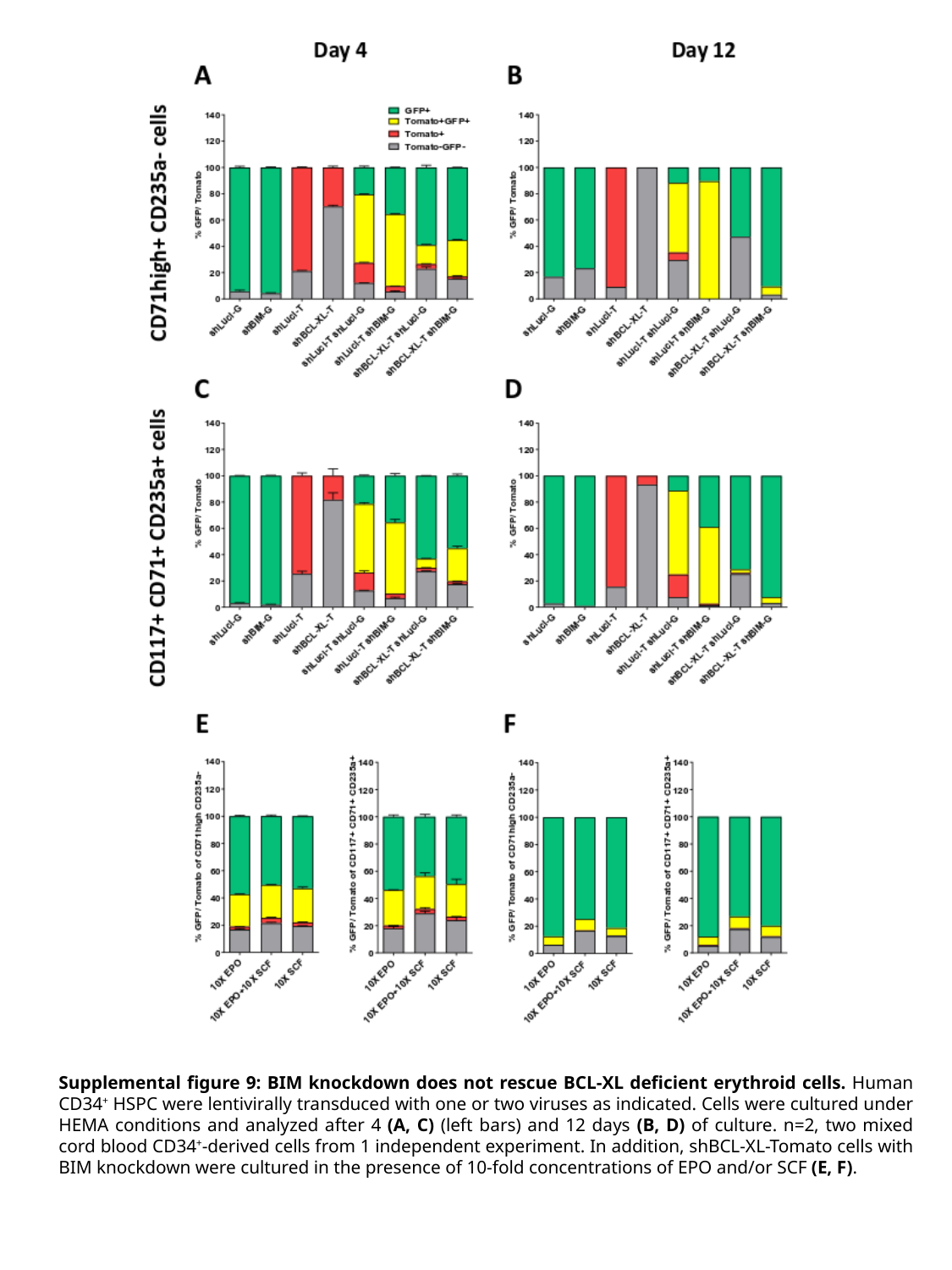

Supplemental figure 9: BIM knockdown does not rescue BCL-XL deficient erythroid cells. Human CD34+ HSPC were lentivirally transduced with one or two viruses as indicated. Cells were cultured under HEMA conditions and analyzed after 4 (A, C) (left bars) and 12 days (B, D) of culture. n=2, two mixed cord blood CD34+-derived cells from 1 independent experiment. In addition, shBCL-XL-Tomato cells with BIM knockdown were cultured in the presence of 10-fold concentrations of EPO and/or SCF (E, F).

## Slide 12
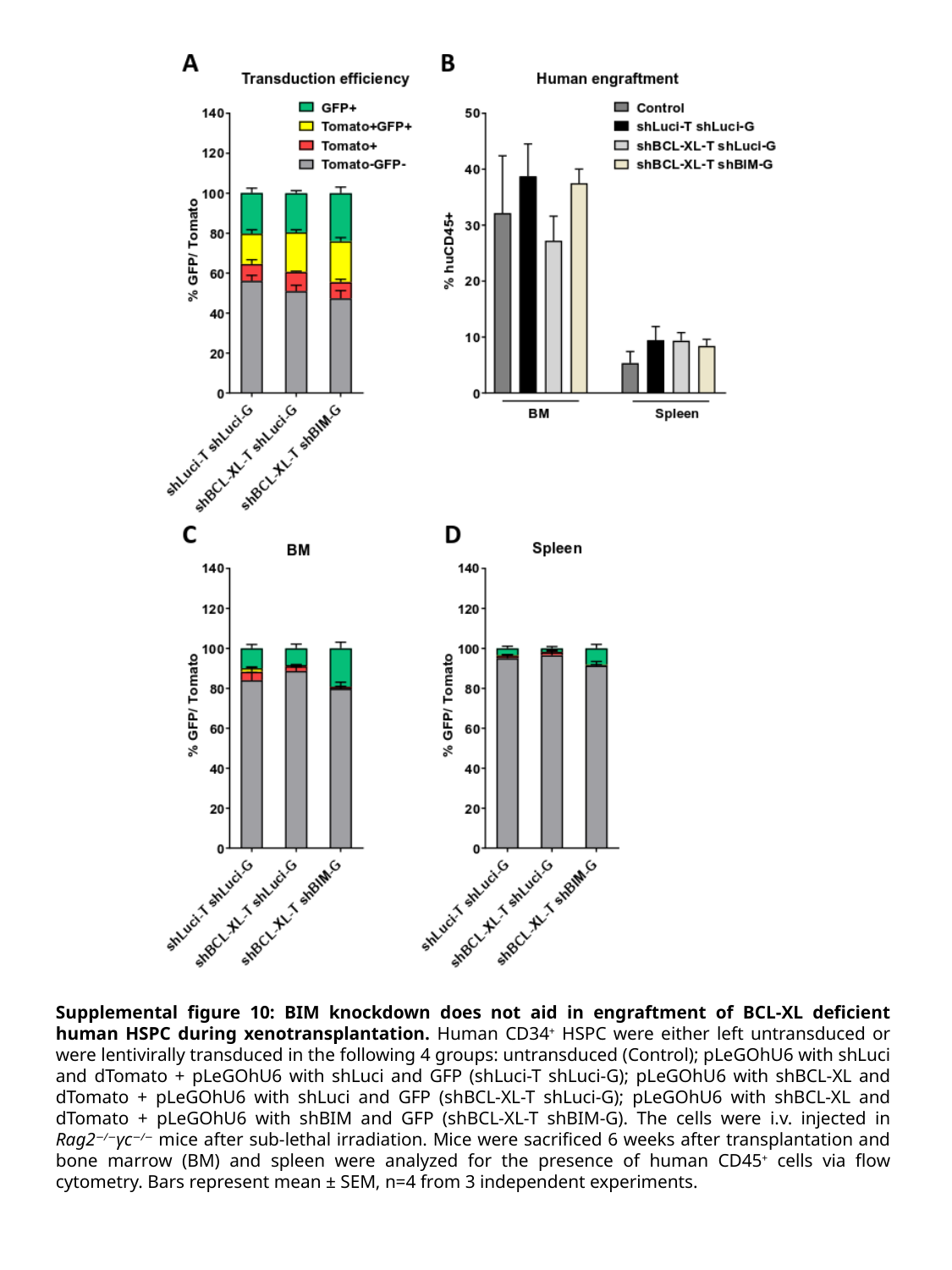

Supplemental figure 10: BIM knockdown does not aid in engraftment of BCL-XL deficient human HSPC during xenotransplantation. Human CD34+ HSPC were either left untransduced or were lentivirally transduced in the following 4 groups: untransduced (Control); pLeGOhU6 with shLuci and dTomato + pLeGOhU6 with shLuci and GFP (shLuci-T shLuci-G); pLeGOhU6 with shBCL-XL and dTomato + pLeGOhU6 with shLuci and GFP (shBCL-XL-T shLuci-G); pLeGOhU6 with shBCL-XL and dTomato + pLeGOhU6 with shBIM and GFP (shBCL-XL-T shBIM-G). The cells were i.v. injected in Rag2−/−γc−/− mice after sub-lethal irradiation. Mice were sacrificed 6 weeks after transplantation and bone marrow (BM) and spleen were analyzed for the presence of human CD45+ cells via flow cytometry. Bars represent mean ± SEM, n=4 from 3 independent experiments.

## Slide 13
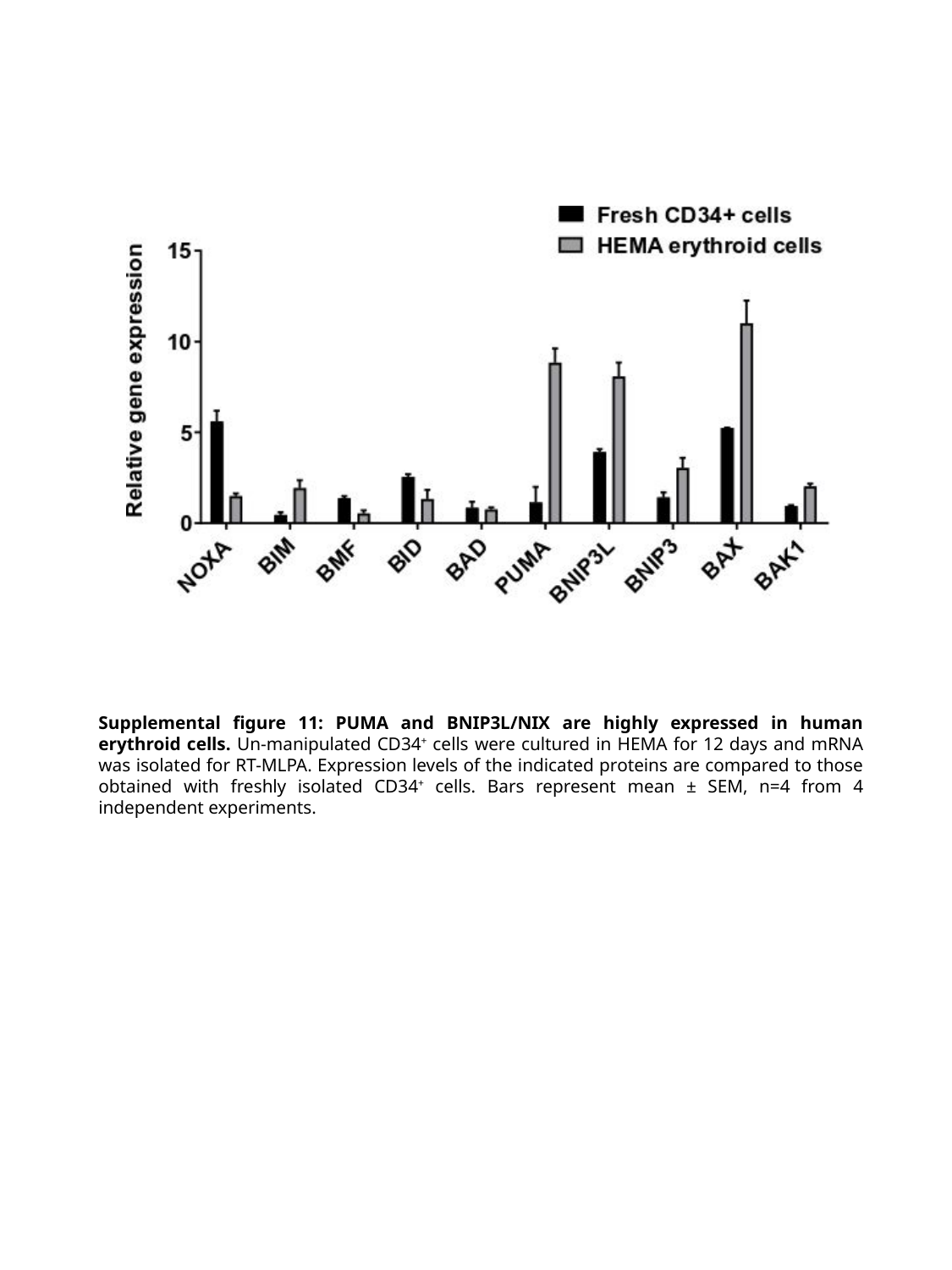

Supplemental figure 11: PUMA and BNIP3L/NIX are highly expressed in human erythroid cells. Un-manipulated CD34+ cells were cultured in HEMA for 12 days and mRNA was isolated for RT-MLPA. Expression levels of the indicated proteins are compared to those obtained with freshly isolated CD34+ cells. Bars represent mean ± SEM, n=4 from 4 independent experiments.
